# Supplementary figures and images for: Deep mutational scanning identifies sites in influenza nucleoprotein that affect viral inhibition by MxA
Source: PLoS Pathog. 2017 Mar 27;13(3):e1006288. doi: 10.1371/journal.ppat.1006288 (PMC5383324; doi:10.1371/journal.ppat.1006288)

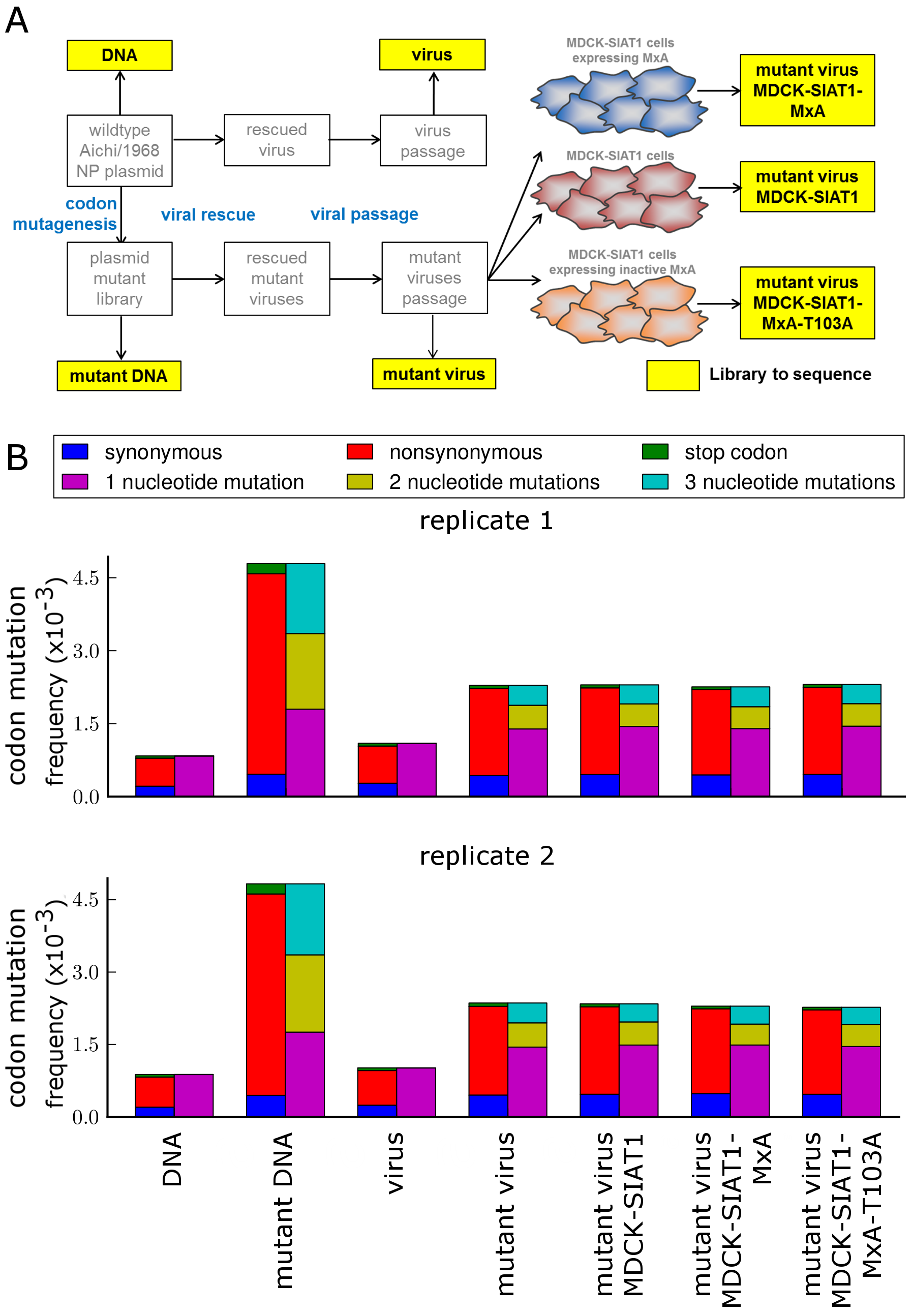

Supplement: S1 Fig — (A) Schematic of samples sequenced. Beginning with a plasmid carrying unmutated NP, we created a library of plasmids encoding codon mutants of NP as described in [32]. We then used reverse genetics to generate wildtype and mutant viruses from unmutated and mutagenized plasmids and passaged these viruses at low MOI in MDCK-SIAT1 cells as described in [32]. We then infected the mutant virus libraries into the three cell lines shown in Fig 1A. (B) The average per-codon mutation frequency for each sample in each library. Codon mutations are classified by the type of mutation or the number of nucleotide changes relative to the wildtype codon. Mutation rates for the mutant virus passaged through MxA-expressing cells and for mutant virus passaged through MxA non-expressing or inactive cells were similar. However this does not preclude interesting MxA selection occurring on a site-by-site basis. Note that the background error rates estimated from sequencing the unmutated DNA and virus are less than the mutation rates in the libraries; in addition, these errors affect only the single-nucleotide codon mutations. (TIF) [file ppat.1006288.s001.tif]

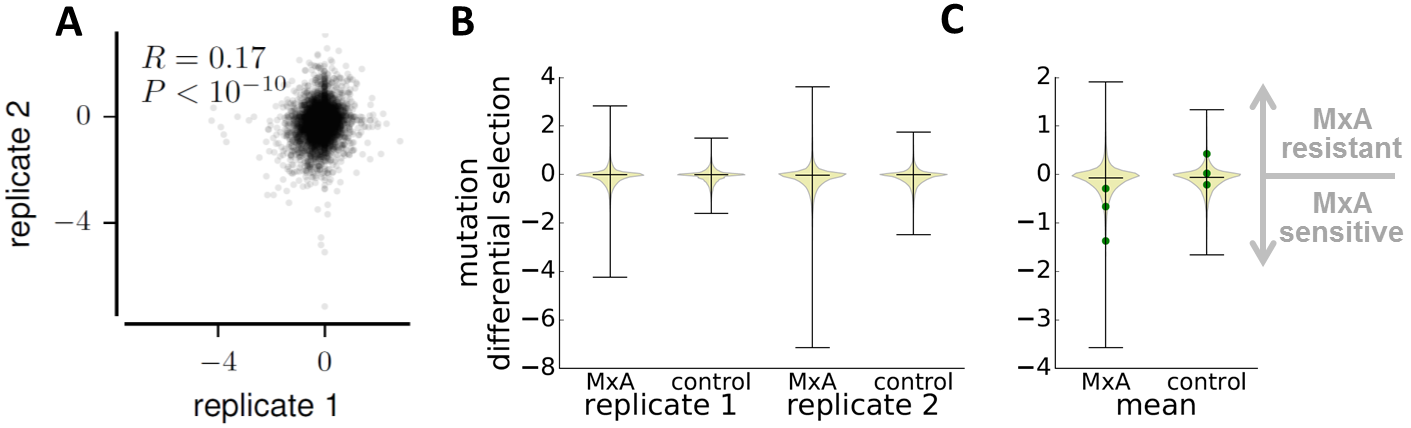

Supplement: S2 Fig — This figure differs from Fig 3 by showing data for individual mutations rather than sites (see Fig 1B for the distinction between mutation and site differential selection). (A) Differential selections for individual mutations are weakly correlated between experimental replicates. The weakness of this correlation relative to that for the site differential selections shown in Fig 3C is likely because at any NP site that is a determinant of MxA resistance, several mutations usually affect MxA resistance. Therefore even if replicates do not evenly sample the same individual mutations at that site [30, 32], both replicates will still detect similar total differential selection when averaged across all mutations at a site. (B) Distributions of differential selection for all mutations. Distributions are displayed for the actual (MDCK-SIAT1-MxA vs MDCK-SIAT1) and control (MDCK-SIAT1-MxA-T103A vs MDCK-SIAT) selections for both replicates of the mutational scanning. The selection distributions have longer tails than the control distributions, which indicates that some mutations affect MxA sensitivity more than the background experimental noise. (C) The distributions of mutation differential selections for the mean of the replicates. The differential selection values for mutations V100R, P283L, and Y313F previously demonstrated [17] to increase MxA sensitivity in 1918 virus are shown in green. Each mutation increased MxA sensitivity in our data as expected. (TIF) [file ppat.1006288.s002.tif]

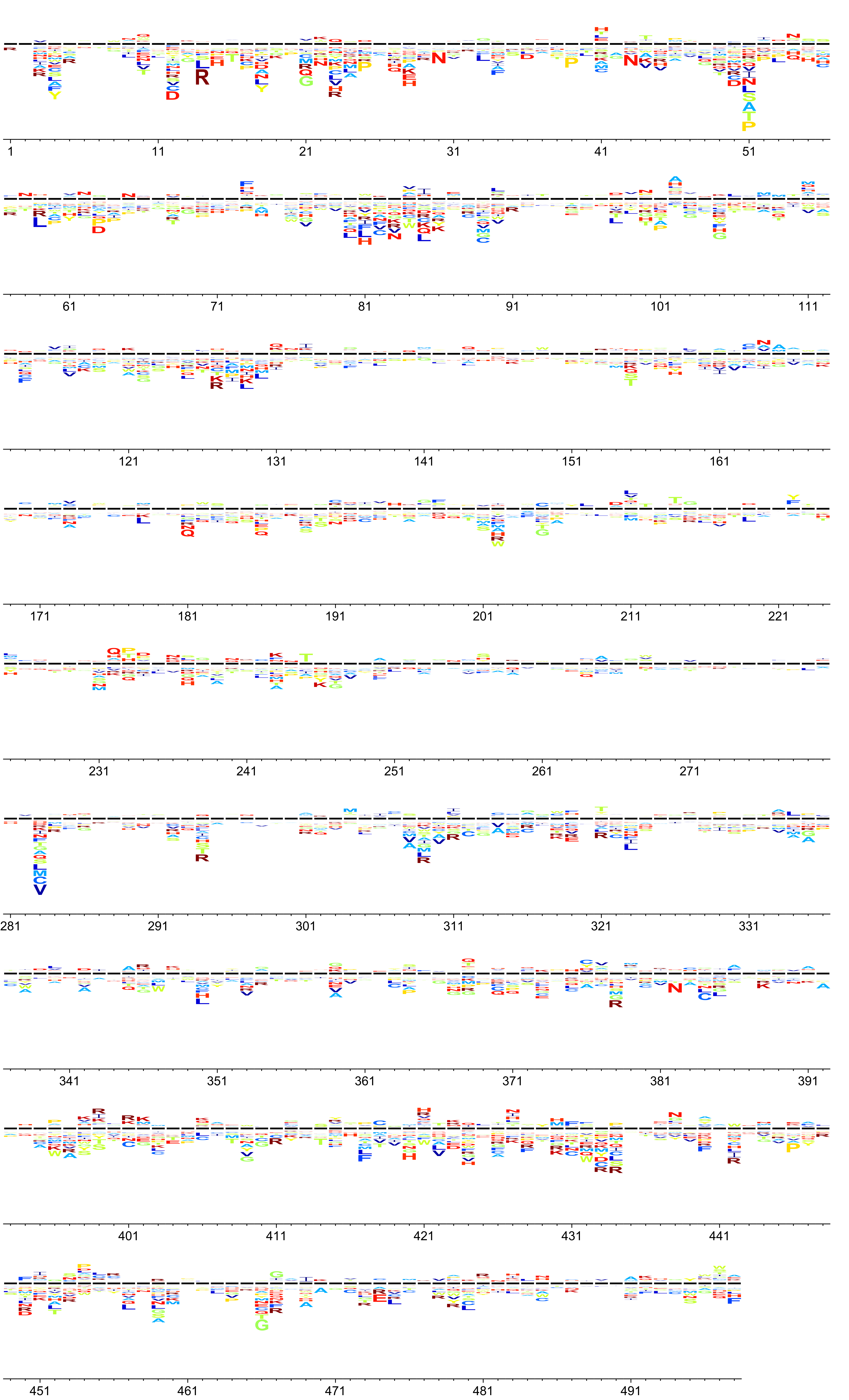

Supplement: S3 Fig — The height of each letter above or below the black center line indicates selection for or against that mutation in MxA-expressing versus non-expressing cells (see Fig 1B). These data are the average of the two replicates, and the values are given in S3 File. Letters are colored by amino-acid hydrophobicity. (TIF) [file ppat.1006288.s003.tif]

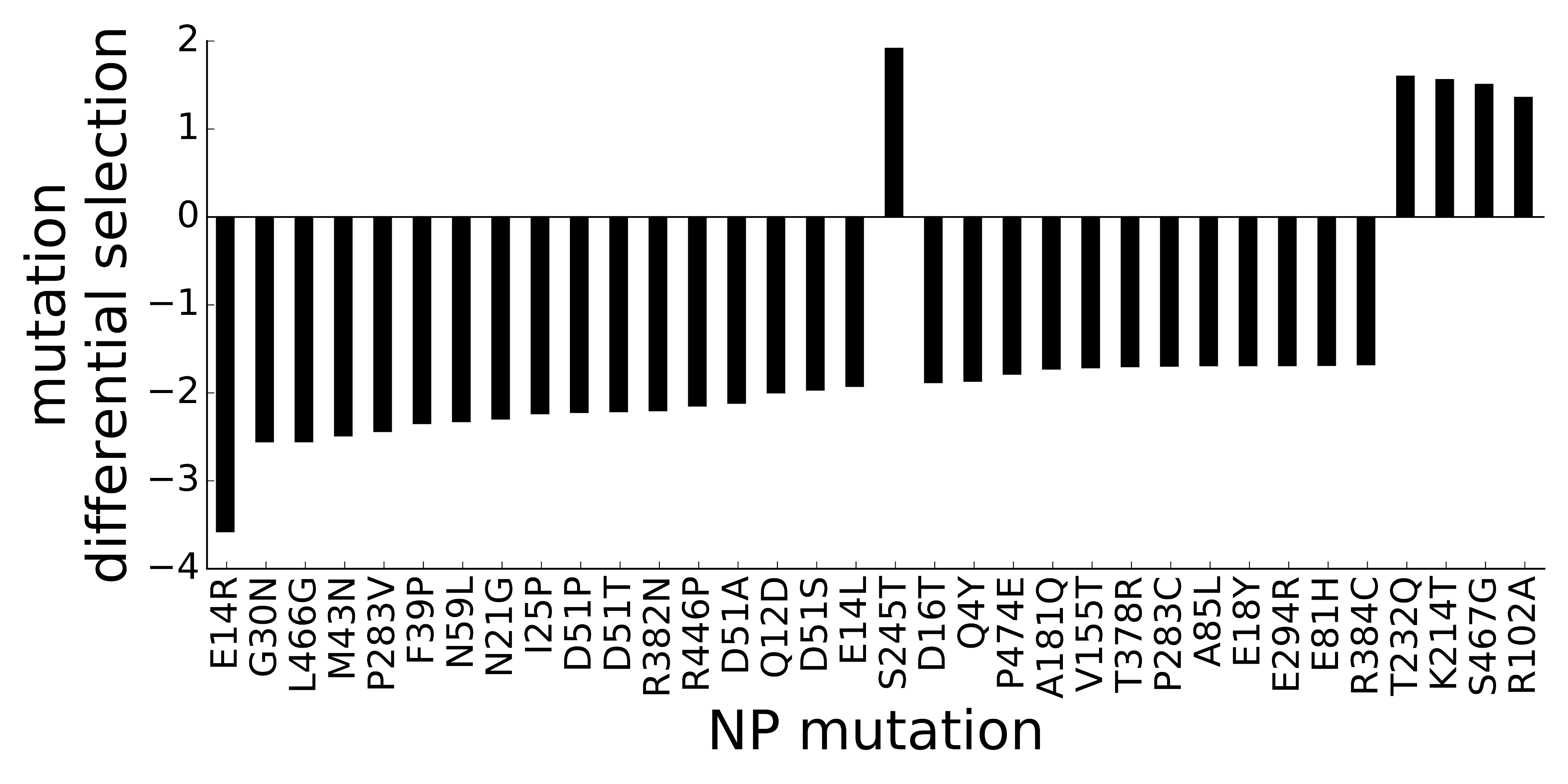

Supplement: S4 Fig — Differential selections for mutations in MxA-expressing vs non-expressing cells after averaging the data for the two replicates. Shown are all individual mutations that have differential selections more extreme than that seen in the control selections: 5 mutations have differential selections greater than the maximum seen in the control selection, and 29 mutations have differential selections less than the minimum seen in the control selection. Most mutations have negative differential selection, and 14 of 34 mutations occur at the sites shown in Fig 4B as having high differential selection. (TIF) [file ppat.1006288.s004.tif]

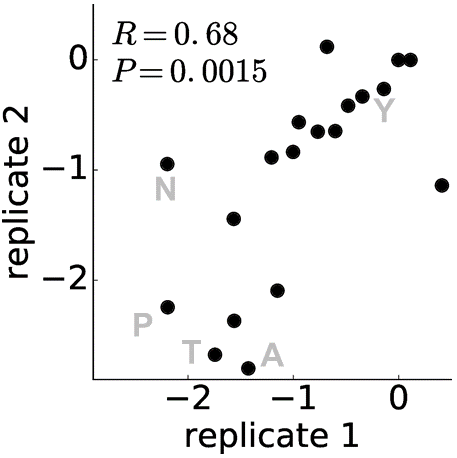

Supplement: S5 Fig — The differential selection for MxA resistance for the mutations at site 51 for both replicates of the deep mutational scanning. A negative value indicates the mutation increases MxA sensitivity. (TIF) [file ppat.1006288.s005.tif]

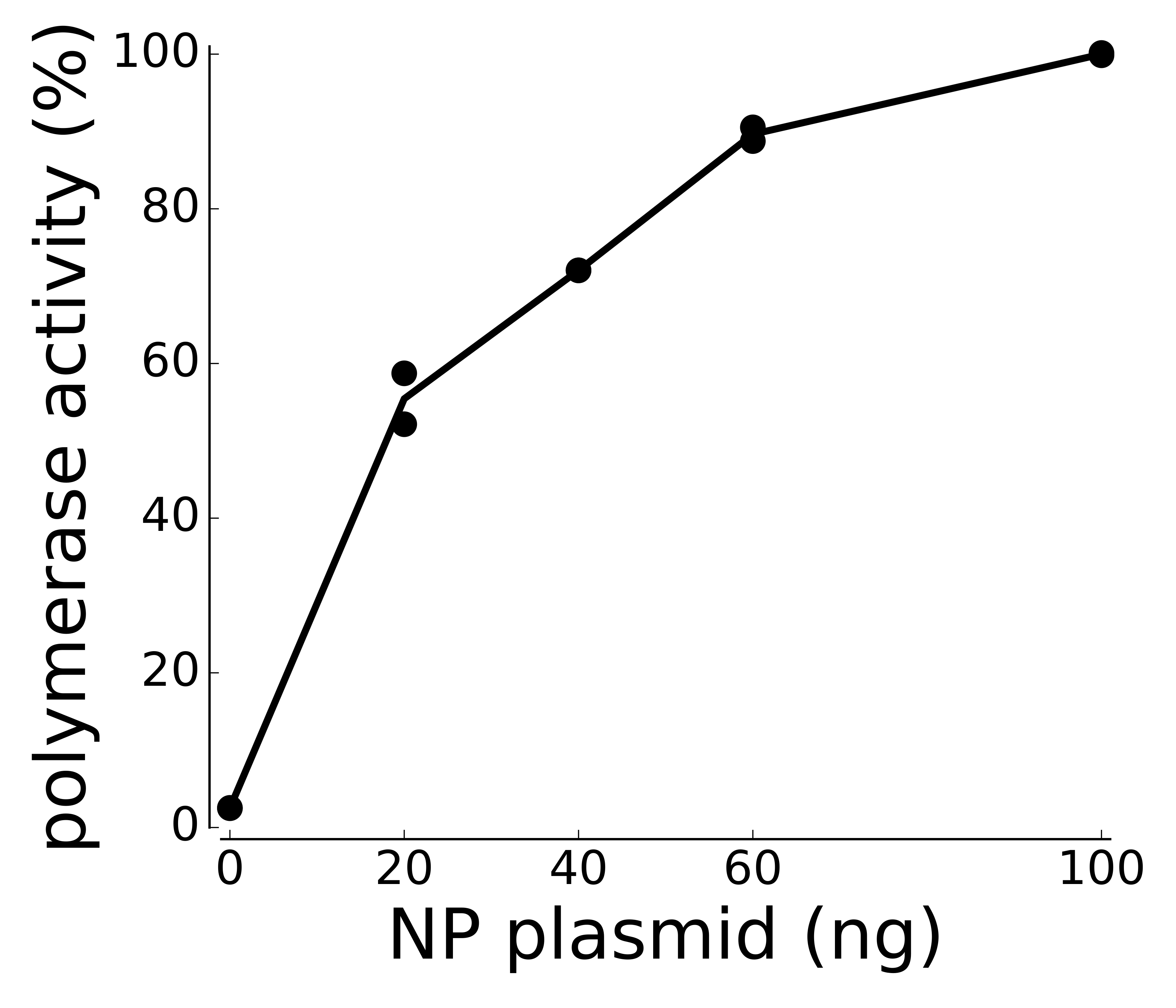

Supplement: S6 Fig — The polymerase activity as a function of the amount of transfected NP plasmid. The Aichi/1968 NP plasmid was varied from 0 to 100 ng, while the PB1, PB2, and PA plasmids were fixed at 125 ng each and the GFP reporter plasmid was fixed at 500 ng. The experiment was done in duplicate and the polymerase activity of the mix with the maximum amount of NP is set to 100%. For all our polymerase activity assays, we chose an amount of NP (15 ng) near the midpoint of the dose-response curve. At this level of NP, the reporter signal is sensitive to changes in the amount of NP due to MxA activity. (TIF) [file ppat.1006288.s006.tif]

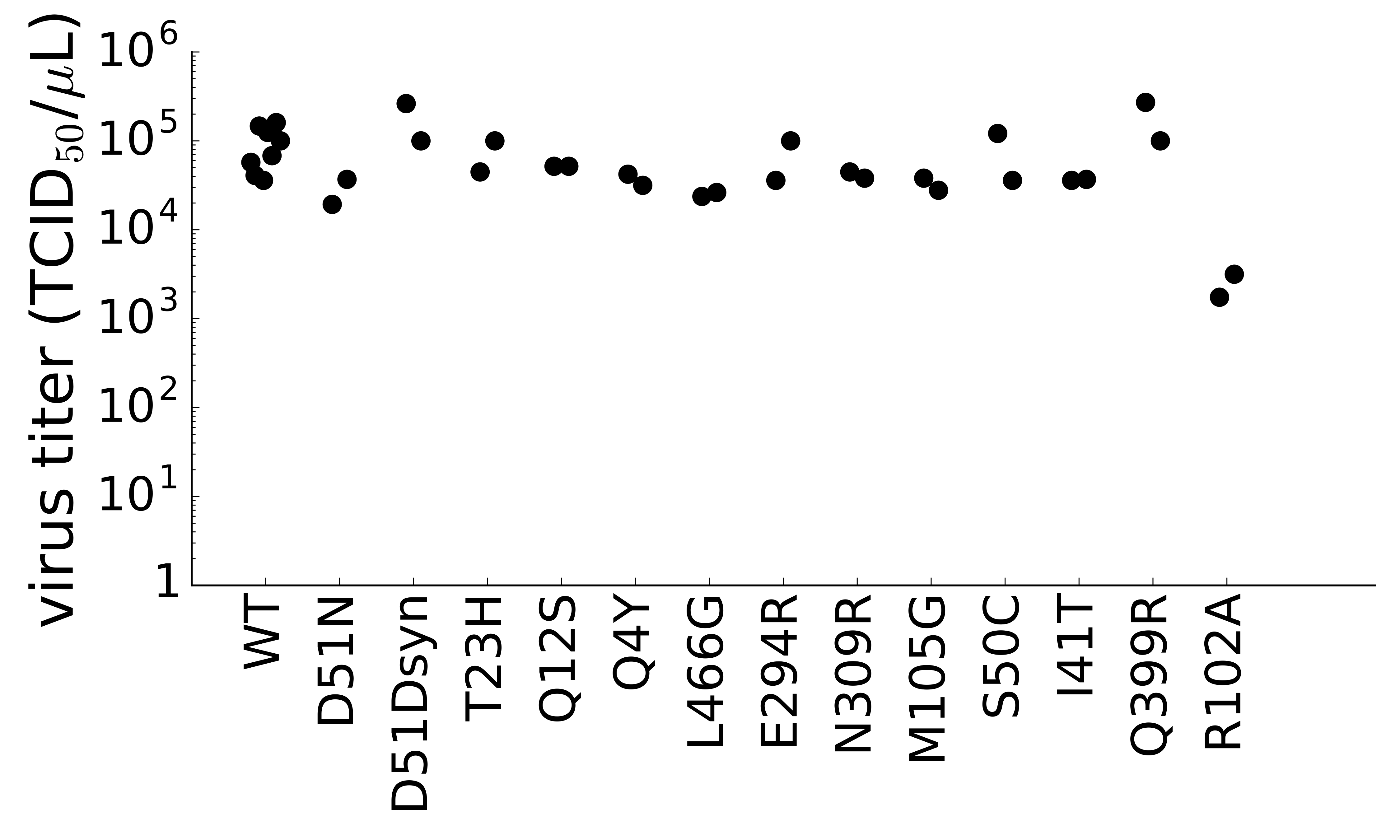

Supplement: S7 Fig — We used reverse genetics to generate viruses carrying the wildtype NP, and the indicated mutations in NP, and measured viral titers after 72 hours. We generated duplicate stocks of each virus. The titer of wildtype virus was measured four times for each duplicate stock, while titers of each mutant virus were measured once for each stock. (TIF) [file ppat.1006288.s007.tif]

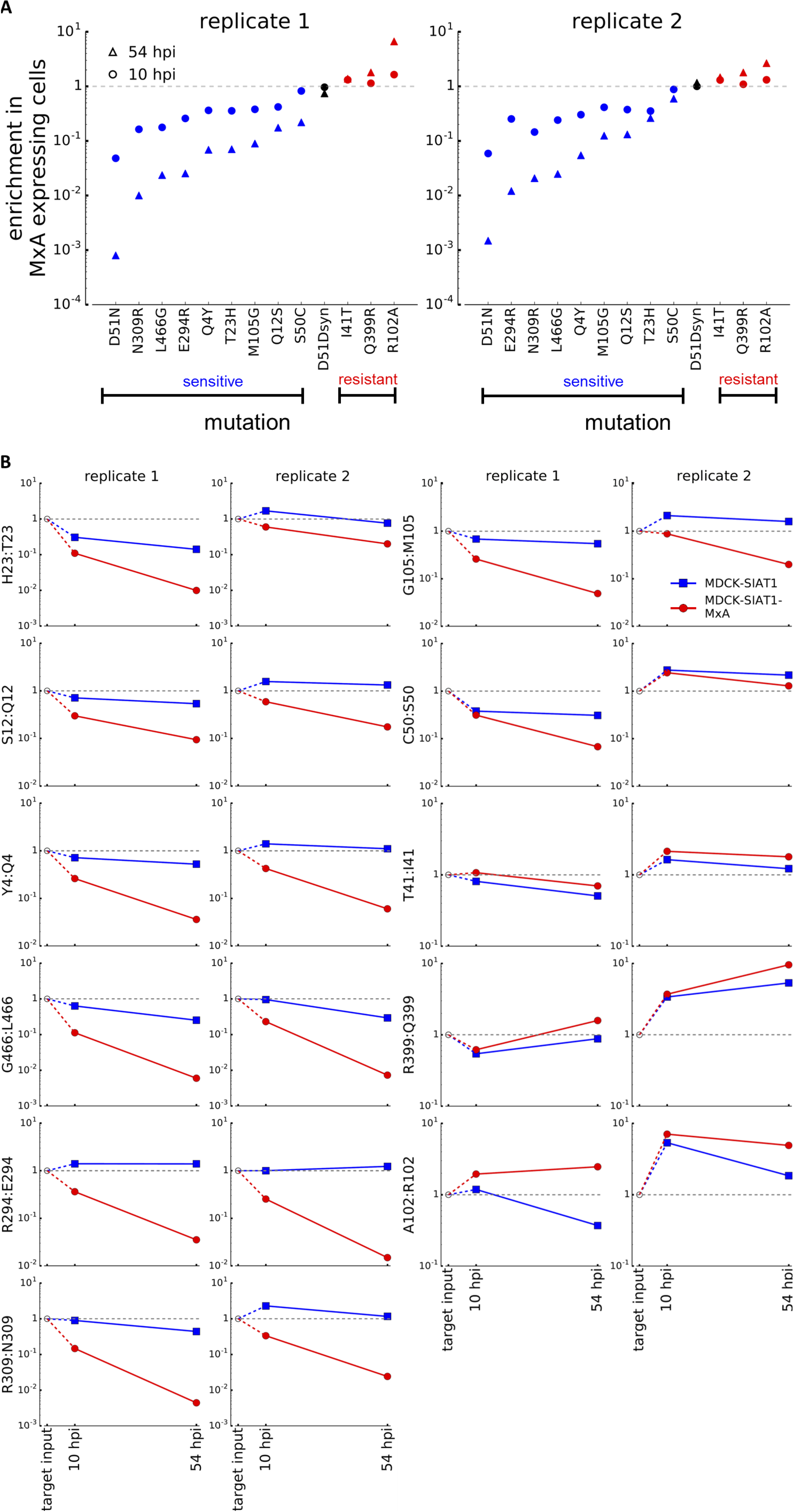

Supplement: S8 Fig — We performed each viral competition experiment in biological replicate and measured the enrichment (A) and relative frequency for each nucleoprotein mutation (B). The average enrichment values across replicates are shown in Fig 7. (TIF) [file ppat.1006288.s008.tif]

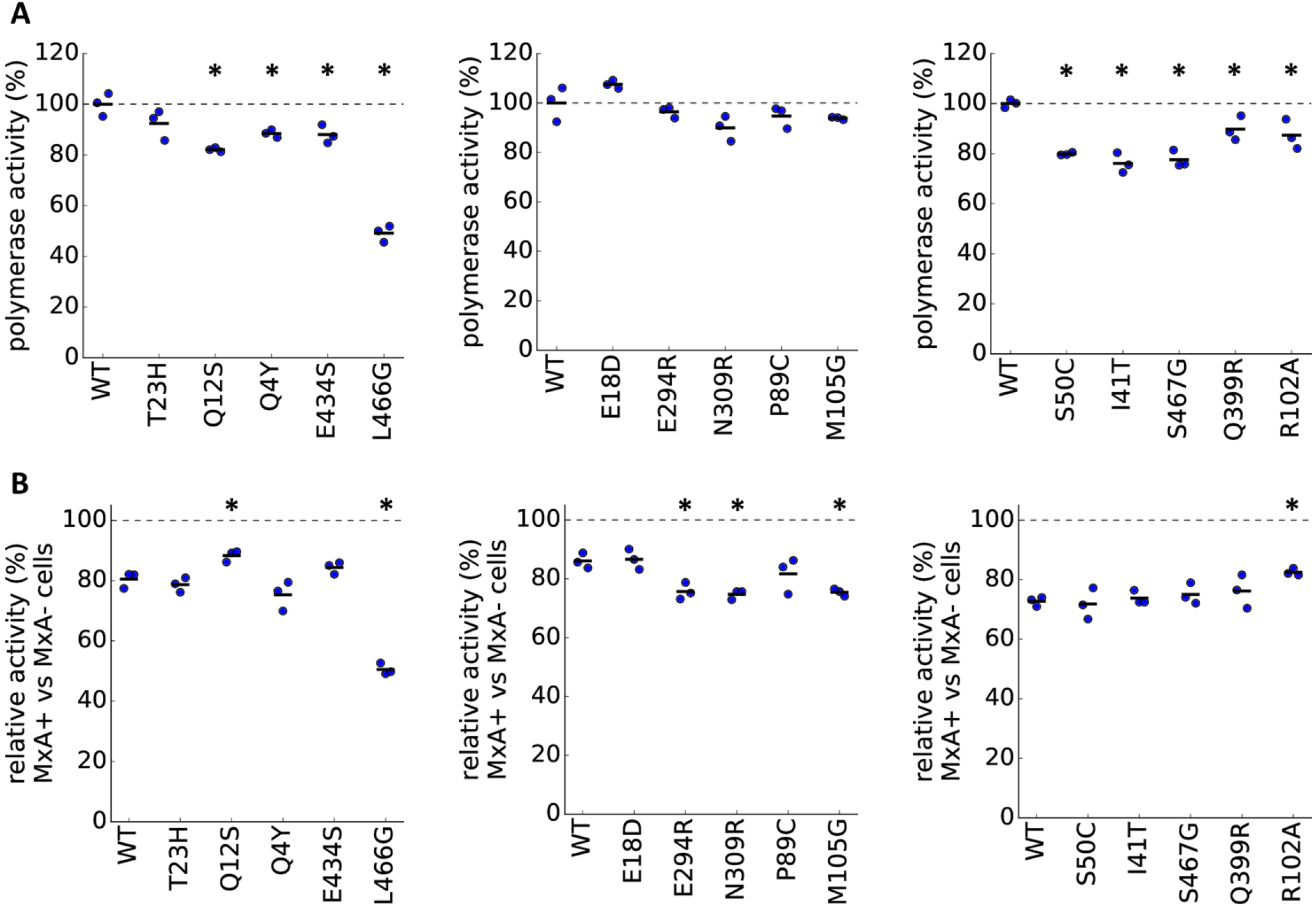

Supplement: S9 Fig — We measured polymerase activity (A) and change in MxA sensitivity (B) for each nucleoprotein mutation using the same approach as in Fig 5A. We used the Student’s t-test to identify which mutant NPs differed significantly (P<0.05) in activity from the wildtype NP, and we marked such sites with *. (TIFF) [file ppat.1006288.s009.tiff]
