# Supplementary material for: Deep mutational scanning identifies sites in influenza nucleoprotein that affect viral inhibition by MxA
Source: PLoS Pathog. 2017 Mar 27;13(3):e1006288. doi: 10.1371/journal.ppat.1006288 (PMC5383324; doi:10.1371/journal.ppat.1006288)
Supplement: S1 Table — For influenza strains from each host, we downloaded all available full-length NP protein sequences from the Influenza Research Database (https://www.fludb.org) (last accessed August 15, 2016). Across all strains, D occurs at site 51 with 99% frequency. (PDF) [file ppat.1006288.s010.pdf]

S1 Table. Amino-acid counts at NP site 51 across influenza hosts.

Host	Amino acids and counts at NP site 51
avian	D 12648, Y 110, E 62, N 22, H 1, S 1, T 1
human	D 9675, E 1, G 1, T 1
swine	D 2782, T 9, Y 3, E 1
equine	D 129
dog	D 115, N 1

For influenza strains from each host, we downloaded all available full-length NP protein sequences from the Influenza Research Database (https://www.fludb.org) (last accessed August 15, 2016). Across all strains, D occurs at site 51 with 99% frequency.
