# Supplementary material for: Deep mutational scanning identifies sites in influenza nucleoprotein that affect viral inhibition by MxA
Source: PLoS Pathog. 2017 Mar 27;13(3):e1006288. doi: 10.1371/journal.ppat.1006288 (PMC5383324; doi:10.1371/journal.ppat.1006288)
Supplement: S1 File — This ZIP file contains the computer code and input data files to perform the analysis described in this paper. (ZIP) [file ppat.1006288.s011.zip › Supplemental_File_1_code/CompetitionNotebook.html]

CompetitionNotebook


# Analysis of sequencing data from competition between wildtype virus and mutant virus¶

Orr Ashenberg, 9 March 2017

To run the commands in this notebook, please first install ```mapmuts``` and ```dms\_tools```, and then verify that they can be run in the directory in which you placed this ipython notebook.

Here, we take sequencing data from each viral competition experiment and calculate the frequency of the wildtype versus the mutant virus. We tested amino-acid mutations and synonymous codon mutations. There are 4 experimental conditions for the amino-acid mutation and 4 for the synonymous mutation:

- CMU: 10 hrs post-infection, collect cellular RNA from MDCK-SIAT1 cells
- CXU: 10 hrs post-infection, collect cellular RNA from MDCK-SIAT1-MxA cells
- CMS: 54 hrs post-infection, collect cellular RNA from MDCK-SIAT1 cells
- CXS: 54 hrs post-infection, collect cellular RNA from MDCK-SIAT1-MxA cells

---

## Table of contents¶

### Load required modules and raw data¶

Preliminary set-up and load required modules

Download sequencing data from the SRA

### Calculate mutant and wildtype codon counts at each NP site¶

Align paired-end reads to NP and count mutations

Calculate mutation frequency at each NP site

### Plot competition-experiment results¶

Plot enrichment of mutation relative to wildtype in MxA-expressing vs non-expressing cells

Plot relative NP mutation frequencies over time

---

## Load required modules and raw data¶

**Preliminary set-up and load required modules:**

In [1]:

```
import os
basedir = os.getcwd()
if not os.path.isdir('notebook_figures'):
    !mkdir notebook_figures
if not os.path.isdir('FASTQ_files'):
    !mkdir FASTQ_files
if not os.path.isdir('FASTQ_files/site51'):
    !mkdir FASTQ_files/site51
if not os.path.isdir('FASTQ_files/additional_sites'):
    !mkdir FASTQ_files/additional_sites
figdir = '%s/notebook_figures' % basedir

import matplotlib
print "Using matplotlib version %s" % matplotlib.__version__
matplotlib.use("Pdf")
import numpy as np
import matplotlib.pyplot as plt
import scipy.stats
import Bio.SeqIO
from operator import itemgetter
import os
import subprocess
import math
import glob
import pandas as pd
pd.options.display.float_format = '{:,.3f}'.format # Limit output to 3 decimal places.
import dms_tools
import dms_tools.file_io
import dms_tools.utils
from runscriptfunctions import * # functions to facilitate running parallel jobs on the Fred Hutch scientific computing cluster using `slurm`
```

```
Using matplotlib version 1.5.0
```

Specifications for running scripts. Nothing here needs to be changed by the user.

In [2]:

```
# Where FASTQ files from SRA will be downloaded
FASTQdir = '%s/FASTQ_files' % basedir

# Specify the biological replicates and library amplicons that were sequenced.
replicates = ['1', '2']

# Specify reference sequence to map reads to, numbering must follow what is specified by alignspecs
refseq = '%s/input/Aichi68-NP_amplicon.fa' % basedir
refseq = [seq for seq in Bio.SeqIO.parse(open(refseq), 'fasta')]
refseq = str(refseq[0].seq).upper()
refseq = refseq[61:1558]   # get full Aichi/68-NP coding sequence from start to stop codon

# Specify the experiments that test viral growth early or late in infection, and in MDCK-SIAT1 or MDCK-SIAT1-MxA cells.
# The experiments are further specified by whether they are competitions between nonsynonymous or synonynous mutants.
experiments_nonsyn = ['CMU', 'CXU', 'CMS', 'CXS']
experiments_syn = ['CMUsyn', 'CXUsyn', 'CMSsyn', 'CXSsyn']
experiments = experiments_nonsyn + experiments_syn

# Specify which NP mutations were tested in the viral competition experiment.
# List of tuples (NP site, wt aa, mutant aa, Bloom lab plasmid number) or (NP site, wt codon, synonymous codon, Bloom lab plasmid number)
tested_muts = [('51', 'D', 'N', 1409), ('51', 'GAT', 'GAC', 1500), ('23', 'T', 'H', 1599), ('12', 'Q', 'S', 1600), ('4', 'Q', 'Y', 1601), 
               ('466', 'L', 'G', 1603), ('294', 'E', 'R', 1605), ('309', 'N', 'R', 1606), ('105', 'M', 'G', 1608), ('50', 'S', 'C', 1609), 
               ('41', 'I', 'T', 1610), ('399', 'Q', 'R', 1612), ('102', 'R', 'A', 1613)] 

# Specify R1 and R2 read lengths and gene range of each NP amplicon, for aligning reads to amplicons. 
# The alignspecs are split into whether they are used for nonsynonymous, amino-acid mutations, or for synonymous, codon mutations.
alignspecs_nonsyn = [(113, 186, 1, 1, 1409), (38, 101, 1, 1, 1599), (6, 64, 1, 1, 1600), (1, 46, 21, 1, 1601), (1375, 1422, 1, 1, 1603), 
                     (856, 917, 1, 1, 1605), (894, 958, 1, 1, 1606), (274, 352, 1, 1, 1608), (112, 177, 1, 1, 1609), (87, 160, 1, 1, 1610), 
                     (1160, 1225, 1, 1, 1612), (270, 340, 1, 1, 1613)]
alignspecs_syn = [(113, 186, 1, 1, 1500)]

# List of amplicons using Bloom lab plasmid numbering
# The amplicons are split into whether they cover nonsynonymous amino-acid mutations, or synonymous codon mutations
amplicons_nonsyn = [amplicon for (a, b, c, d, amplicon) in alignspecs_nonsyn]   
amplicons_syn = [amplicon for (a, b, c, d, amplicon) in alignspecs_syn]

# Link Bloom lab plasmid amplicon numbering to Aichi/68 NP site numbering
amplicon_to_site = dict([ (amplicon, site) for (site, y, z, amplicon) in tested_muts])   

# Specify quality parameters (Q score thresholds) for deciding whether to use a given R1 and R2 read pair, and for how many nt mutations to allow while aligning amplicons
minq = 15
maxlowqfrac = 0.075
maxmuts = 2 

# Specify whether FASTQ sequence files are gzipped 
gzipped = False
```

**Download sequencing data from the SRA:**

Sequencing data comes from two independent competition experiments that tested different sets of NP mutations. One set of reads covers NP site 51 and the second set covers all other NP sites.

In [3]:

```
%%capture

# All NP sites in competition other than site 51
samples = {'SRR5314799':'CXS_additional-2',
           'SRR5314800':'CMS_additional-2',
           'SRR5314801':'CXU_additional-2',
           'SRR5314802':'CMU_additional-2',
           'SRR5314803':'CXS_additional-1',
           'SRR5314804':'CMS_additional-1',
           'SRR5314805':'CXU_additional-1',
           'SRR5314806':'CMU_additional-1'}

for (accession, samplename) in samples.items():
    print "Downloading and converting file format for sample %s, which corresponds to accession %s." % (samplename, accession)
    !cd FASTQ_files/additional_sites; wget --no-clobber 'ftp://ftp-trace.ncbi.nih.gov/sra/sra-instant/reads/ByRun/sra/SRR/SRR531/'$accession'/'$accession'.sra'
    !mv "FASTQ_files/additional_sites/"$accession".sra" "FASTQ_files/additional_sites/"$samplename".sra"
    !fastq-dump -O FASTQ_files/additional_sites --split-files "FASTQ_files/additional_sites/"$samplename".sra"
    
# NP site 51 in competition 
samples = {'SRR4048747':'CMU-1',
           'SRR4048748':'CXU-1',
           'SRR4048749':'CMS-1',
           'SRR4048750':'CXS-1',
           'SRR4048751':'CMUsyn-1',
           'SRR4048752':'CXUsyn-1',
           'SRR4048753':'CMSsyn-1',
           'SRR4048754':'CXSsyn-1',
           'SRR4048755':'CMU-2',
           'SRR4048756':'CXU-2',
           'SRR4048757':'CMS-2',
           'SRR4048758':'CXS-2',
           'SRR4048759':'CMUsyn-2',
           'SRR4048760':'CXUsyn-2',
           'SRR4048761':'CMSsyn-2',
           'SRR4048762':'CXSsyn-2'}

for (accession, samplename) in samples.items():
    print "Downloading and converting file format for sample %s, which corresponds to accession %s." % (samplename, accession)
    !cd FASTQ_files/site51; wget --no-clobber 'ftp://ftp-trace.ncbi.nih.gov/sra/sra-instant/reads/ByRun/sra/SRR/SRR404/'$accession'/'$accession'.sra'
    !mv "FASTQ_files/site51/"$accession".sra" "FASTQ_files/site51/"$samplename".sra"
    !fastq-dump -O FASTQ_files/site51 --split-files "FASTQ_files/site51/"$samplename".sra"
```

## Calculate mutant and wildtype codon counts at each NP site¶

**Align paired-end reads to NP and count mutations**

Below, we use functions from `dms_tools` to align paired-end reads to amplicons that cover each NP site.

First, we read in the names of read pair FASTQ files for each experiment. Each experimental condition has an R1 read file and an R2 read file. We read in a set of reads for NP site 51 and a set of reads for all other NP sites.

In [4]:

```
# Store (r1file, r2file) in dictionary keyed by (replicate, experiment) ie (1, CMU)

# All NP sites in competition other than site 51
fastqfiles = {}
for replicate in replicates:
    if not os.path.isdir('%s/replicate-%s' % (basedir, replicate)):
        os.mkdir('%s/replicate-%s' % (basedir, replicate))  
    for experiment in experiments_nonsyn:
        if not os.path.isdir('%s/replicate-%s/%s' % (basedir, replicate, experiment)):
            os.mkdir('%s/replicate-%s/%s' % (basedir, replicate, experiment))
        r1file = '%s/additional_sites/%s_additional-%s_1.fastq' % (FASTQdir, experiment, replicate)
        r2file = '%s/additional_sites/%s_additional-%s_2.fastq' % (FASTQdir, experiment, replicate)
        fastqfiles[(replicate, experiment)] = (r1file, r2file)     

# NP site 51 in competition 
fastqfiles_site51 = {}
for replicate in replicates:
    if not os.path.isdir('%s/replicate-%s' % (basedir, replicate)):
        os.mkdir('%s/replicate-%s' % (basedir, replicate))  
    for experiment in experiments:
        if not os.path.isdir('%s/replicate-%s/%s' % (basedir, replicate, experiment)):
            os.mkdir('%s/replicate-%s/%s' % (basedir, replicate, experiment))
        r1file_site51 = '%s/site51/%s-%s_1.fastq' % (FASTQdir, experiment, replicate)
        r2file_site51 = '%s/site51/%s-%s_2.fastq' % (FASTQdir, experiment, replicate)
        fastqfiles_site51[(replicate, experiment)] = (r1file_site51, r2file_site51)
```

Next we iterate through R1, R2 read pairs for each experiment and check quality of reads. Read pairs are discarded if any read has above a certain fraction of nucleotides with low Q score.

In [5]:

```
# Keep count of how many reads fall into each of the below categories
readcategories = ['total read pairs', 'read pairs that fail Illumina filter', 'low quality read pairs']

# All NP sites in competition other than site 51
# Dictionary keyed by 'replicate experiment' ie (1 CMU), values are 2-tuple of paired R1 and R2 reads that pass quality filters
readpairs = {}

for replicate in replicates:
    for experiment in experiments_nonsyn: 
        readpairs['%s %s' % (replicate, experiment)] = []     
        
        # Dictionary keeping count of what category each read pair falls into 
        n = dict([(category, 0) for category in readcategories])
        
        (r1file, r2file) = fastqfiles[(replicate, experiment)]
        print r1file, r2file

        for read_tup in dms_tools.file_io.IteratePairedFASTQ([r1file], [r2file], gzipped, applyfilter=True):
            n['total read pairs'] += 1

            if read_tup:   # Read pair passed Illumina chastity filter
                (name, r1, r2, q1, q2) = read_tup
                (r1, r2, q1, q2) = dms_tools.utils.TrimReads(r1, r2, q1, q2, 46, 46)
                qcheckedreads = dms_tools.utils.CheckReadQuality(r1, r2, q1, q2, minq, maxlowqfrac, barcodelength=0)
                if qcheckedreads: # Read pair does not have a high fraction of ambiguous N nucleotides
                    (r1, r2) = qcheckedreads
                    readpairs['%s %s' % (replicate, experiment)].append((r1, r2))
                else:
                    n['low quality read pairs'] += 1
            else:
                n['read pairs that fail Illumina filter'] += 1
            if n['total read pairs'] % 1e5 == 0:
                print 'Reads parsed in %s %s so far: %d' % (replicate, experiment, n['total read pairs'])
                
                
# NP site 51 in competition 
readpairs_site51 = {}

for replicate in replicates:
    for experiment in experiments: 
        readpairs_site51['%s %s' % (replicate, experiment)] = []     
        
        # Dictionary keeping count of what category each read pair falls into 
        n = dict([(category, 0) for category in readcategories])

        (r1file, r2file) = fastqfiles_site51[(replicate, experiment)]
        print r1file, r2file

        for read_tup in dms_tools.file_io.IteratePairedFASTQ([r1file], [r2file], gzipped, applyfilter=True):
            n['total read pairs'] += 1

            if read_tup:   # Read pair passed Illumina chastity filter
                (name, r1, r2, q1, q2) = read_tup
                (r1, r2, q1, q2) = dms_tools.utils.TrimReads(r1, r2, q1, q2, 46, 46)
                qcheckedreads = dms_tools.utils.CheckReadQuality(r1, r2, q1, q2, minq, maxlowqfrac, barcodelength=0)
                if qcheckedreads: # Read pair does not have a high fraction of ambiguous N nucleotides
                    (r1, r2) = qcheckedreads
                    readpairs_site51['%s %s' % (replicate, experiment)].append((r1, r2))
                else:
                    n['low quality read pairs'] += 1
            else:
                n['read pairs that fail Illumina filter'] += 1
            if n['total read pairs'] % 1e5 == 0:
                print 'Reads parsed in %s %s so far: %d' % (replicate, experiment, n['total read pairs'])
```

```
/fh/fast/bloom_j/grp/oashenbe/2016_NP_MxA_paper/FASTQ_files/additional_sites/CMU_additional-1_1.fastq /fh/fast/bloom_j/grp/oashenbe/2016_NP_MxA_paper/FASTQ_files/additional_sites/CMU_additional-1_2.fastq
Reads parsed in 1 CMU so far: 100000
/fh/fast/bloom_j/grp/oashenbe/2016_NP_MxA_paper/FASTQ_files/additional_sites/CXU_additional-1_1.fastq /fh/fast/bloom_j/grp/oashenbe/2016_NP_MxA_paper/FASTQ_files/additional_sites/CXU_additional-1_2.fastq
Reads parsed in 1 CXU so far: 100000
Reads parsed in 1 CXU so far: 200000
/fh/fast/bloom_j/grp/oashenbe/2016_NP_MxA_paper/FASTQ_files/additional_sites/CMS_additional-1_1.fastq /fh/fast/bloom_j/grp/oashenbe/2016_NP_MxA_paper/FASTQ_files/additional_sites/CMS_additional-1_2.fastq
Reads parsed in 1 CMS so far: 100000
Reads parsed in 1 CMS so far: 200000
/fh/fast/bloom_j/grp/oashenbe/2016_NP_MxA_paper/FASTQ_files/additional_sites/CXS_additional-1_1.fastq /fh/fast/bloom_j/grp/oashenbe/2016_NP_MxA_paper/FASTQ_files/additional_sites/CXS_additional-1_2.fastq
Reads parsed in 1 CXS so far: 100000
Reads parsed in 1 CXS so far: 200000
/fh/fast/bloom_j/grp/oashenbe/2016_NP_MxA_paper/FASTQ_files/additional_sites/CMU_additional-2_1.fastq /fh/fast/bloom_j/grp/oashenbe/2016_NP_MxA_paper/FASTQ_files/additional_sites/CMU_additional-2_2.fastq
Reads parsed in 2 CMU so far: 100000
/fh/fast/bloom_j/grp/oashenbe/2016_NP_MxA_paper/FASTQ_files/additional_sites/CXU_additional-2_1.fastq /fh/fast/bloom_j/grp/oashenbe/2016_NP_MxA_paper/FASTQ_files/additional_sites/CXU_additional-2_2.fastq
Reads parsed in 2 CXU so far: 100000
/fh/fast/bloom_j/grp/oashenbe/2016_NP_MxA_paper/FASTQ_files/additional_sites/CMS_additional-2_1.fastq /fh/fast/bloom_j/grp/oashenbe/2016_NP_MxA_paper/FASTQ_files/additional_sites/CMS_additional-2_2.fastq
Reads parsed in 2 CMS so far: 100000
/fh/fast/bloom_j/grp/oashenbe/2016_NP_MxA_paper/FASTQ_files/additional_sites/CXS_additional-2_1.fastq /fh/fast/bloom_j/grp/oashenbe/2016_NP_MxA_paper/FASTQ_files/additional_sites/CXS_additional-2_2.fastq
Reads parsed in 2 CXS so far: 100000
/fh/fast/bloom_j/grp/oashenbe/2016_NP_MxA_paper/FASTQ_files/site51/CMU-1_1.fastq /fh/fast/bloom_j/grp/oashenbe/2016_NP_MxA_paper/FASTQ_files/site51/CMU-1_2.fastq
Reads parsed in 1 CMU so far: 100000
/fh/fast/bloom_j/grp/oashenbe/2016_NP_MxA_paper/FASTQ_files/site51/CXU-1_1.fastq /fh/fast/bloom_j/grp/oashenbe/2016_NP_MxA_paper/FASTQ_files/site51/CXU-1_2.fastq
Reads parsed in 1 CXU so far: 100000
/fh/fast/bloom_j/grp/oashenbe/2016_NP_MxA_paper/FASTQ_files/site51/CMS-1_1.fastq /fh/fast/bloom_j/grp/oashenbe/2016_NP_MxA_paper/FASTQ_files/site51/CMS-1_2.fastq
Reads parsed in 1 CMS so far: 100000
/fh/fast/bloom_j/grp/oashenbe/2016_NP_MxA_paper/FASTQ_files/site51/CXS-1_1.fastq /fh/fast/bloom_j/grp/oashenbe/2016_NP_MxA_paper/FASTQ_files/site51/CXS-1_2.fastq
Reads parsed in 1 CXS so far: 100000
/fh/fast/bloom_j/grp/oashenbe/2016_NP_MxA_paper/FASTQ_files/site51/CMUsyn-1_1.fastq /fh/fast/bloom_j/grp/oashenbe/2016_NP_MxA_paper/FASTQ_files/site51/CMUsyn-1_2.fastq
/fh/fast/bloom_j/grp/oashenbe/2016_NP_MxA_paper/FASTQ_files/site51/CXUsyn-1_1.fastq /fh/fast/bloom_j/grp/oashenbe/2016_NP_MxA_paper/FASTQ_files/site51/CXUsyn-1_2.fastq
/fh/fast/bloom_j/grp/oashenbe/2016_NP_MxA_paper/FASTQ_files/site51/CMSsyn-1_1.fastq /fh/fast/bloom_j/grp/oashenbe/2016_NP_MxA_paper/FASTQ_files/site51/CMSsyn-1_2.fastq
/fh/fast/bloom_j/grp/oashenbe/2016_NP_MxA_paper/FASTQ_files/site51/CXSsyn-1_1.fastq /fh/fast/bloom_j/grp/oashenbe/2016_NP_MxA_paper/FASTQ_files/site51/CXSsyn-1_2.fastq
/fh/fast/bloom_j/grp/oashenbe/2016_NP_MxA_paper/FASTQ_files/site51/CMU-2_1.fastq /fh/fast/bloom_j/grp/oashenbe/2016_NP_MxA_paper/FASTQ_files/site51/CMU-2_2.fastq
Reads parsed in 2 CMU so far: 100000
Reads parsed in 2 CMU so far: 200000
/fh/fast/bloom_j/grp/oashenbe/2016_NP_MxA_paper/FASTQ_files/site51/CXU-2_1.fastq /fh/fast/bloom_j/grp/oashenbe/2016_NP_MxA_paper/FASTQ_files/site51/CXU-2_2.fastq
Reads parsed in 2 CXU so far: 100000
Reads parsed in 2 CXU so far: 200000
/fh/fast/bloom_j/grp/oashenbe/2016_NP_MxA_paper/FASTQ_files/site51/CMS-2_1.fastq /fh/fast/bloom_j/grp/oashenbe/2016_NP_MxA_paper/FASTQ_files/site51/CMS-2_2.fastq
Reads parsed in 2 CMS so far: 100000
/fh/fast/bloom_j/grp/oashenbe/2016_NP_MxA_paper/FASTQ_files/site51/CXS-2_1.fastq /fh/fast/bloom_j/grp/oashenbe/2016_NP_MxA_paper/FASTQ_files/site51/CXS-2_2.fastq
Reads parsed in 2 CXS so far: 100000
Reads parsed in 2 CXS so far: 200000
/fh/fast/bloom_j/grp/oashenbe/2016_NP_MxA_paper/FASTQ_files/site51/CMUsyn-2_1.fastq /fh/fast/bloom_j/grp/oashenbe/2016_NP_MxA_paper/FASTQ_files/site51/CMUsyn-2_2.fastq
/fh/fast/bloom_j/grp/oashenbe/2016_NP_MxA_paper/FASTQ_files/site51/CXUsyn-2_1.fastq /fh/fast/bloom_j/grp/oashenbe/2016_NP_MxA_paper/FASTQ_files/site51/CXUsyn-2_2.fastq
/fh/fast/bloom_j/grp/oashenbe/2016_NP_MxA_paper/FASTQ_files/site51/CMSsyn-2_1.fastq /fh/fast/bloom_j/grp/oashenbe/2016_NP_MxA_paper/FASTQ_files/site51/CMSsyn-2_2.fastq
/fh/fast/bloom_j/grp/oashenbe/2016_NP_MxA_paper/FASTQ_files/site51/CXSsyn-2_1.fastq /fh/fast/bloom_j/grp/oashenbe/2016_NP_MxA_paper/FASTQ_files/site51/CXSsyn-2_2.fastq
```

Below, for each read pair in each experiment, try to align reads to one of the possible NP amplicons. Each NP amplicon is the product of a 1st and 2nd round PCR reaction centered around the mutation site of interest. The reactions add the Illumina adapters and experimental barcodes. A different alignspecs for aligning reads to an amplicon is used depending on whether the experiment is testing a nonysynonymous amino-acid mutation or a synonymous codon mutation. The counts for each experiment and mutation site combination are written to codon counts and amino-acid counts files.

In [6]:

```
# Iterate over all read pairs from each experiment, and align the reads to an amplicon region of the NP reference sequence.
# If the read pair aligns, update the mutation counts.
# All NP sites in competition other than site 51
for replicate in replicates:
    for experiment in experiments_nonsyn:         
        # Choose alignspecs and amplicons based on whether this competition experiment is testing a nonsynonymous amino-acid mutation or synonymous codon mutation.
        alignspecs = alignspecs_nonsyn[1:]
        amplicons = amplicons_nonsyn[1:]
            
        # Initialize codon count dictionary for each amplicon to which the reads may be aligned
        # Key is amplicon and value is codon count dictionary that holds mutation counts in format for dms_tools.file_io.WriteDMSCounts
        counts = {}
        for amplicon in amplicons:
            counts[amplicon] = {}
            for r in range(1, len(refseq) // 3 + 1):
                counts[amplicon][str(r)] = dict([('WT', refseq[3 * r - 3 : 3 * r])] + [(codon, 0) for codon in dms_tools.codons])

        # For each read pair, try to align reads to amplicon iterating over the different amplicons specified by alignspecs
        numalign = 0
        for (r1, r2) in readpairs['%s %s' % (replicate, experiment)]:
            for (refseqstart, refseqend, r1start, r2start, amplicon) in alignspecs:
                maxN = int(maxlowqfrac * min(refseqend - refseqstart + 1, len(r1) + len(r2) - r1start - r2start + 2))
                if r1start >= len(r1) or r2start >= len(r2):
                    aligned = False # reads too short to align
                else:
                    aligned = dms_tools.utils.AlignSubamplicon(refseq, r1[r1start - 1 : ], r2[r2start - 1 : ], refseqstart, refseqend, maxmuts, 
                                                               maxN, 'codon', counts[amplicon], use_cutils=True)
                if aligned:
                    numalign += 1
                    break   # found the amplicon to which these reads align
                            
        # Write the codon and amino acid counts for this experimental condition and set of amplicons (experiment, replicate, amplicon)
        countsdir = '%s/replicate-%s/%s' % (basedir, replicate, experiment)
        if not os.path.isdir(countsdir):
            os.mkdir(countsdir)
        for amplicon in amplicons:
            codoncountsfile = '%s/%s_%s_%s_codoncounts.txt' % (countsdir, replicate, experiment, amplicon)
            aacountsfile = '%s/%s_%s_%s_aacounts.txt' % (countsdir, replicate, experiment, amplicon)
            dms_tools.file_io.WriteDMSCounts(codoncountsfile, counts[amplicon])
            
            # Translate codon counts to amino-acid counts and write to file
            aacounts = dms_tools.file_io.ReadDMSCounts(codoncountsfile, 'codon', translate_codon_to_aa=True)
            dms_tools.file_io.WriteDMSCounts(aacountsfile, aacounts)
            
            
# NP site 51 in competition 
for replicate in replicates:
    for experiment in experiments:         
        # Choose alignspecs and amplicons based on whether this competition experiment is testing a nonsynonymous amino-acid mutation or synonymous codon mutation.
        if experiment in experiments_syn:
            alignspecs = [alignspecs_syn[0]]
            amplicons = [amplicons_syn[0]]
        else:
            alignspecs = [alignspecs_nonsyn[0]]
            amplicons = [amplicons_nonsyn[0]]
            
        # Initialize codon count dictionary for each amplicon to which the reads may be aligned
        # Key is amplicon and value is codon count dictionary that holds mutation counts in format for dms_tools.file_io.WriteDMSCounts
        counts = {}
        for amplicon in amplicons:
            counts[amplicon] = {}
            for r in range(1, len(refseq) // 3 + 1):
                counts[amplicon][str(r)] = dict([('WT', refseq[3 * r - 3 : 3 * r])] + [(codon, 0) for codon in dms_tools.codons])

        # For each read pair, try to align reads to amplicon iterating over the different amplicons specified by alignspecs
        numalign = 0
        for (r1, r2) in readpairs_site51['%s %s' % (replicate, experiment)]:
            for (refseqstart, refseqend, r1start, r2start, amplicon) in alignspecs:
                maxN = int(maxlowqfrac * min(refseqend - refseqstart + 1, len(r1) + len(r2) - r1start - r2start + 2))
                if r1start >= len(r1) or r2start >= len(r2):
                    aligned = False # reads too short to align
                else:
                    aligned = dms_tools.utils.AlignSubamplicon(refseq, r1[r1start - 1 : ], r2[r2start - 1 : ], refseqstart, refseqend, maxmuts, 
                                                               maxN, 'codon', counts[amplicon], use_cutils=True)
                if aligned:
                    numalign += 1
                    break   # found the amplicon to which these reads align
                            
        # Write the codon and amino acid counts for this experimental condition and set of amplicons (experiment, replicate, amplicon)
        countsdir = '%s/replicate-%s/%s' % (basedir, replicate, experiment)
        if not os.path.isdir(countsdir):
            os.mkdir(countsdir)
        for amplicon in amplicons:
            codoncountsfile = '%s/%s_%s_%s_codoncounts.txt' % (countsdir, replicate, experiment, amplicon)
            aacountsfile = '%s/%s_%s_%s_aacounts.txt' % (countsdir, replicate, experiment, amplicon)
            dms_tools.file_io.WriteDMSCounts(codoncountsfile, counts[amplicon])
            
            # Translate codon counts to amino-acid counts and write to file
            aacounts = dms_tools.file_io.ReadDMSCounts(codoncountsfile, 'codon', translate_codon_to_aa=True)
            dms_tools.file_io.WriteDMSCounts(aacountsfile, aacounts)
```

**Calculate mutation frequency at each NP site**

The function below calculates amino acid or codon frequencies at a specified site

In [7]:

```
def CharacterFrequencyAtSite(counts, site, chartype):
    '''This function takes a dictionary of amino-acid or codon counts and calculates the frequency
    of each amino acid or codon at a specified site.
    
    *counts* : Dictionary of amino-acid or codon counts for each site using the format in `dms_tools.file_io.ReadDMSCounts`
    *site* : String giving the site at which to calculate amino-acid or codon frequencies of each amino acid or codon
    *chartype* is the type of character. Valid values are the following strings:
        - *codon* : DNA codons, those listed in the *codons* variable in `dms_tools.file_io'
        - *aminoacids_nostop* : amino acids, not including stop codons.
        - *aminoacids_withstop* : amino acids, including stop codons (``*``).
    *freqs* : Dictionary where each key is an amino acid or codon and the value is the corresponding
    amino-acid or codon frequency at that **site**
    '''
    if chartype.upper() == 'CODON':
        characters = dms_tools.codons
    elif chartype.upper() == 'AMINOACIDS_NOSTOP':
        characters = dms_tools.aminoacids_nostop
    elif chartype.upper() == 'AMINOACIDS_WITHSTOP':
        characters = dms_tools.aminoacids_withstop
    else:
        raise ValueError("Invalid chartype of %s" % chartype)
    if site not in counts:
        raise ValueError('Specified site %s not found in counts dictionary' % site)
                         
    totalcounts = sum([counts[site][char] for char in characters])
    if totalcounts:
        freqs = dict( [(char, float(counts[site][char]) / totalcounts) for char in characters] )
    else:
        print 'There are no counts at site %s' % site,
        freqs = dict( [(char, 0) for char in characters] )
    return freqs
```

Next we read in the codon counts and amino-acid counts at each NP site for each competition experiment and calculate their mutation frequencies.

In [8]:

```
# Dictionaries storing amino-acid or codon frequencies. The key is 'replicate, experiment, site' ie (1, CMU, 51) and the value is
# a dictionary keyed by amino-acid or codon and the value is the corresponding frequency.
aafreqs = {}
codonfreqs = {}

# Read in amino-acid counts and codon counts, and calculate their frequencies at the sites tested in the competition experiment
for replicate in replicates:
    for experiment in experiments: 
        countsdir = '%s/replicate-%s/%s' % (basedir, replicate, experiment)
        # Choose amplicons based on whether this competition experiment is testing a nonsynonymous (amino-acid mutation) or synonymous codon mutation
        if experiment in experiments_syn:
            amplicons = amplicons_syn
        else:
            amplicons = amplicons_nonsyn
            
        for amplicon in amplicons:
            site = amplicon_to_site[amplicon]        # NP site in competition experiment

            aacountsfile = '%s/%s_%s_%s_aacounts.txt' % (countsdir, replicate, experiment, amplicon)
            aacounts = dms_tools.file_io.ReadDMSCounts(aacountsfile, 'aminoacids_withstop')
            aafreqs['%s %s %s' % (replicate, experiment, site)] = CharacterFrequencyAtSite(aacounts, site, 'aminoacids_withstop')
            
            codoncountsfile = '%s/%s_%s_%s_codoncounts.txt' % (countsdir, replicate, experiment, amplicon)
            codoncounts = dms_tools.file_io.ReadDMSCounts(codoncountsfile, 'codon')
            codonfreqs['%s %s %s' % (replicate, experiment, site)] = CharacterFrequencyAtSite(codoncounts, site, 'codon')
```

Next we print out amino-acid or codon mutation frequencies for the mutations tested in the competition experiment. We also quantify how each mutation was enriched (mutant amino acid relative to wildtype amino acid, or synonymous codon relative to wildtype codon). This enrichment, or relative mutation frequency, gets plotted at the end of the notebook.

In [9]:

```
columns = ['site', 'replicate', 'experiment', 'wt', 'mut', 'wt_freq', 'mut_freq']
freq = pd.DataFrame(columns=columns)

for replicate in replicates:
    for (site, char1, char2, amplicon) in tested_muts:        
        # If this tested mutation is an amino-acid mutation
        if amplicon in amplicons_nonsyn:      
            (wt, mut) = (char1, char2)
            for experiment in experiments_nonsyn:
                expkey = '%s %s %s' % (replicate, experiment, site)
                freq = freq.append(pd.DataFrame([[site, replicate, experiment, wt, mut, aafreqs[expkey][wt], aafreqs[expkey][mut]]], columns=columns))

        # If this tested mutation is a synonymous mutation
        elif amplicon in amplicons_syn:
            (wt, mut) = (char1, char2)
            for experiment in experiments_syn:
                expkey = '%s %s %s' % (replicate, experiment, site)
                freq = freq.append(pd.DataFrame([[site, replicate, experiment, wt, mut, codonfreqs[expkey][wt], codonfreqs[expkey][mut]]], columns=columns))
        else:
            raise ValueError('Amplicon %s not found' % amplicon)
        
# Use mutation frequencies to calculate relative frequency of each mutation relative to wildtype
freq['mut_wt_ratio'] = freq['mut_freq']/freq['wt_freq']   
print freq[freq.replicate=='1'], '\n', freq[freq.replicate=='2']
```

```
  site replicate experiment   wt  mut  wt_freq  mut_freq  mut_wt_ratio
0   51         1        CMU    D    N    0.334     0.665         1.989
0   51         1        CXU    D    N    0.912     0.087         0.096
0   51         1        CMS    D    N    0.449     0.551         1.227
0   51         1        CXS    D    N    0.999     0.001         0.001
0   51         1     CMUsyn  GAT  GAC    0.532     0.468         0.880
0   51         1     CXUsyn  GAT  GAC    0.541     0.459         0.850
0   51         1     CMSsyn  GAT  GAC    0.586     0.414         0.707
0   51         1     CXSsyn  GAT  GAC    0.655     0.344         0.526
0   23         1        CMU    T    H    0.764     0.235         0.308
0   23         1        CXU    T    H    0.901     0.099         0.110
0   23         1        CMS    T    H    0.876     0.124         0.141
0   23         1        CXS    T    H    0.990     0.010         0.010
0   12         1        CMU    Q    S    0.584     0.415         0.711
0   12         1        CXU    Q    S    0.769     0.230         0.299
0   12         1        CMS    Q    S    0.650     0.349         0.537
0   12         1        CXS    Q    S    0.913     0.086         0.094
0    4         1        CMU    Q    Y    0.582     0.418         0.718
0    4         1        CXU    Q    Y    0.793     0.207         0.261
0    4         1        CMS    Q    Y    0.656     0.344         0.524
0    4         1        CXS    Q    Y    0.965     0.035         0.036
0  466         1        CMU    L    G    0.612     0.388         0.634
0  466         1        CXU    L    G    0.898     0.101         0.112
0  466         1        CMS    L    G    0.798     0.201         0.252
0  466         1        CXS    L    G    0.994     0.006         0.006
0  294         1        CMU    E    R    0.416     0.583         1.400
0  294         1        CXU    E    R    0.734     0.266         0.362
0  294         1        CMS    E    R    0.418     0.582         1.392
0  294         1        CXS    E    R    0.965     0.034         0.035
0  309         1        CMU    N    R    0.526     0.472         0.898
0  309         1        CXU    N    R    0.871     0.128         0.147
0  309         1        CMS    N    R    0.693     0.306         0.441
0  309         1        CXS    N    R    0.995     0.004         0.004
0  105         1        CMU    M    G    0.594     0.405         0.683
0  105         1        CXU    M    G    0.793     0.206         0.259
0  105         1        CMS    M    G    0.646     0.353         0.546
0  105         1        CXS    M    G    0.953     0.047         0.049
0   50         1        CMU    S    C    0.724     0.275         0.380
0   50         1        CXU    S    C    0.761     0.238         0.313
0   50         1        CMS    S    C    0.763     0.236         0.309
0   50         1        CXS    S    C    0.936     0.063         0.068
0   41         1        CMU    I    T    0.552     0.448         0.811
0   41         1        CXU    I    T    0.482     0.517         1.072
0   41         1        CMS    I    T    0.663     0.336         0.507
0   41         1        CXS    I    T    0.588     0.411         0.699
0  399         1        CMU    Q    R    0.648     0.351         0.542
0  399         1        CXU    Q    R    0.618     0.382         0.617
0  399         1        CMS    Q    R    0.532     0.468         0.880
0  399         1        CXS    Q    R    0.386     0.613         1.585
0  102         1        CMU    R    A    0.457     0.542         1.186
0  102         1        CXU    R    A    0.339     0.660         1.949
0  102         1        CMS    R    A    0.729     0.270         0.370
0  102         1        CXS    R    A    0.288     0.711         2.467 
  site replicate experiment   wt  mut  wt_freq  mut_freq  mut_wt_ratio
0   51         2        CMU    D    N    0.251     0.749         2.980
0   51         2        CXU    D    N    0.850     0.149         0.176
0   51         2        CMS    D    N    0.421     0.579         1.374
0   51         2        CXS    D    N    0.998     0.002         0.002
0   51         2     CMUsyn  GAT  GAC    0.737     0.263         0.356
0   51         2     CXUsyn  GAT  GAC    0.737     0.263         0.357
0   51         2     CMSsyn  GAT  GAC    0.786     0.214         0.272
0   51         2     CXSsyn  GAT  GAC    0.759     0.241         0.318
0   23         2        CMU    T    H    0.371     0.628         1.693
0   23         2        CXU    T    H    0.625     0.374         0.599
0   23         2        CMS    T    H    0.566     0.433         0.766
0   23         2        CXS    T    H    0.832     0.167         0.201
0   12         2        CMU    Q    S    0.388     0.611         1.572
0   12         2        CXU    Q    S    0.628     0.371         0.591
0   12         2        CMS    Q    S    0.430     0.570         1.327
0   12         2        CXS    Q    S    0.851     0.149         0.175
0    4         2        CMU    Q    Y    0.416     0.583         1.401
0    4         2        CXU    Q    Y    0.702     0.298         0.425
0    4         2        CMS    Q    Y    0.474     0.525         1.106
0    4         2        CXS    Q    Y    0.943     0.057         0.060
0  466         2        CMU    L    G    0.511     0.488         0.956
0  466         2        CXU    L    G    0.813     0.187         0.231
0  466         2        CMS    L    G    0.772     0.227         0.294
0  466         2        CXS    L    G    0.993     0.007         0.007
0  294         2        CMU    E    R    0.499     0.500         1.002
0  294         2        CXU    E    R    0.797     0.202         0.254
0  294         2        CMS    E    R    0.447     0.552         1.234
0  294         2        CXS    E    R    0.985     0.015         0.015
0  309         2        CMU    N    R    0.303     0.696         2.300
0  309         2        CXU    N    R    0.748     0.251         0.335
0  309         2        CMS    N    R    0.461     0.538         1.166
0  309         2        CXS    N    R    0.976     0.024         0.024
0  105         2        CMU    M    G    0.320     0.680         2.127
0  105         2        CXU    M    G    0.532     0.468         0.880
0  105         2        CMS    M    G    0.385     0.615         1.598
0  105         2        CXS    M    G    0.833     0.166         0.199
0   50         2        CMU    S    C    0.265     0.734         2.767
0   50         2        CXU    S    C    0.291     0.708         2.435
0   50         2        CMS    S    C    0.315     0.684         2.169
0   50         2        CXS    S    C    0.437     0.562         1.288
0   41         2        CMU    I    T    0.380     0.620         1.633
0   41         2        CXU    I    T    0.319     0.681         2.137
0   41         2        CMS    I    T    0.450     0.549         1.220
0   41         2        CXS    I    T    0.358     0.641         1.790
0  399         2        CMU    Q    R    0.228     0.772         3.390
0  399         2        CXU    Q    R    0.212     0.787         3.704
0  399         2        CMS    Q    R    0.158     0.842         5.331
0  399         2        CXS    Q    R    0.095     0.905         9.570
0  102         2        CMU    R    A    0.157     0.842         5.362
0  102         2        CXU    R    A    0.124     0.875         7.069
0  102         2        CMS    R    A    0.351     0.648         1.848
0  102         2        CXS    R    A    0.169     0.830         4.917
```

Next we take the average of the mutation frequencies across replicates, and calculate the resulting relative mutation frequency.

In [10]:

```
columns = ['site', 'experiment', 'wt', 'mut', 'wt_freq', 'mut_freq']
meanfreq = pd.DataFrame(columns=columns)

for (site, wt, mut, amplicon) in tested_muts:  

    # If this tested mutation is a nonsynonymous mutation        
    if amplicon in amplicons_nonsyn:      
        for exp in experiments_nonsyn:
            meanwt = np.mean([float(freq[(freq.site==site) & (freq.replicate==replicate) & (freq.experiment==exp)]['wt_freq']) for replicate in replicates])
            meanmut = np.mean([float(freq[(freq.site==site) & (freq.replicate==replicate) & (freq.experiment==exp)]['mut_freq']) for replicate in replicates])
            meanfreq = meanfreq.append(pd.DataFrame([[site, exp, wt, mut, meanwt, meanmut]], columns=columns))

    # If this tested mutation is a synonymous mutation
    elif amplicon in amplicons_syn:
        for exp in experiments_syn:
            meanwt = np.mean([float(freq[(freq.site==site) & (freq.replicate==replicate) & (freq.experiment==exp)]['wt_freq']) for replicate in replicates])
            meanmut = np.mean([float(freq[(freq.site==site) & (freq.replicate==replicate) & (freq.experiment==exp)]['mut_freq']) for replicate in replicates])
            meanfreq = meanfreq.append(pd.DataFrame([[site, exp, wt, mut, meanwt, meanmut]], columns=columns))

    else:
        raise ValueError('Amplicon %s not found' % amplicon)

# Use mutation frequencies to calculate relative frequency of each mutation relative to wildtype
meanfreq['mut_wt_ratio'] = meanfreq['mut_freq']/meanfreq['wt_freq']   
meanfreq
```

Out[10]:

|  | site | experiment | wt | mut | wt\_freq | mut\_freq | mut\_wt\_ratio |
| --- | --- | --- | --- | --- | --- | --- | --- |
| 0 | 51 | CMU | D | N | 0.293 | 0.707 | 2.414 |
| 0 | 51 | CXU | D | N | 0.881 | 0.118 | 0.134 |
| 0 | 51 | CMS | D | N | 0.435 | 0.565 | 1.298 |
| 0 | 51 | CXS | D | N | 0.998 | 0.002 | 0.002 |
| 0 | 51 | CMUsyn | GAT | GAC | 0.634 | 0.365 | 0.576 |
| 0 | 51 | CXUsyn | GAT | GAC | 0.639 | 0.361 | 0.566 |
| 0 | 51 | CMSsyn | GAT | GAC | 0.686 | 0.314 | 0.458 |
| 0 | 51 | CXSsyn | GAT | GAC | 0.707 | 0.293 | 0.414 |
| 0 | 23 | CMU | T | H | 0.568 | 0.432 | 0.760 |
| 0 | 23 | CXU | T | H | 0.763 | 0.237 | 0.310 |
| 0 | 23 | CMS | T | H | 0.721 | 0.279 | 0.386 |
| 0 | 23 | CXS | T | H | 0.911 | 0.089 | 0.097 |
| 0 | 12 | CMU | Q | S | 0.486 | 0.513 | 1.055 |
| 0 | 12 | CXU | Q | S | 0.699 | 0.301 | 0.430 |
| 0 | 12 | CMS | Q | S | 0.540 | 0.460 | 0.851 |
| 0 | 12 | CXS | Q | S | 0.882 | 0.117 | 0.133 |
| 0 | 4 | CMU | Q | Y | 0.499 | 0.500 | 1.003 |
| 0 | 4 | CXU | Q | Y | 0.747 | 0.252 | 0.338 |
| 0 | 4 | CMS | Q | Y | 0.565 | 0.434 | 0.768 |
| 0 | 4 | CXS | Q | Y | 0.954 | 0.046 | 0.048 |
| 0 | 466 | CMU | L | G | 0.561 | 0.438 | 0.780 |
| 0 | 466 | CXU | L | G | 0.856 | 0.144 | 0.169 |
| 0 | 466 | CMS | L | G | 0.785 | 0.214 | 0.272 |
| 0 | 466 | CXS | L | G | 0.993 | 0.007 | 0.007 |
| 0 | 294 | CMU | E | R | 0.458 | 0.542 | 1.183 |
| 0 | 294 | CXU | E | R | 0.766 | 0.234 | 0.306 |
| 0 | 294 | CMS | E | R | 0.433 | 0.567 | 1.310 |
| 0 | 294 | CXS | E | R | 0.975 | 0.024 | 0.025 |
| 0 | 309 | CMU | N | R | 0.414 | 0.584 | 1.410 |
| 0 | 309 | CXU | N | R | 0.810 | 0.189 | 0.234 |
| 0 | 309 | CMS | N | R | 0.577 | 0.422 | 0.731 |
| 0 | 309 | CXS | N | R | 0.986 | 0.014 | 0.014 |
| 0 | 105 | CMU | M | G | 0.457 | 0.542 | 1.188 |
| 0 | 105 | CXU | M | G | 0.662 | 0.337 | 0.508 |
| 0 | 105 | CMS | M | G | 0.515 | 0.484 | 0.939 |
| 0 | 105 | CXS | M | G | 0.893 | 0.106 | 0.119 |
| 0 | 50 | CMU | S | C | 0.495 | 0.505 | 1.020 |
| 0 | 50 | CXU | S | C | 0.526 | 0.473 | 0.899 |
| 0 | 50 | CMS | S | C | 0.539 | 0.460 | 0.853 |
| 0 | 50 | CXS | S | C | 0.686 | 0.313 | 0.456 |
| 0 | 41 | CMU | I | T | 0.466 | 0.534 | 1.146 |
| 0 | 41 | CXU | I | T | 0.401 | 0.599 | 1.496 |
| 0 | 41 | CMS | I | T | 0.557 | 0.443 | 0.795 |
| 0 | 41 | CXS | I | T | 0.473 | 0.526 | 1.112 |
| 0 | 399 | CMU | Q | R | 0.438 | 0.562 | 1.282 |
| 0 | 399 | CXU | Q | R | 0.415 | 0.584 | 1.407 |
| 0 | 399 | CMS | Q | R | 0.345 | 0.655 | 1.900 |
| 0 | 399 | CXS | Q | R | 0.241 | 0.759 | 3.155 |
| 0 | 102 | CMU | R | A | 0.307 | 0.692 | 2.255 |
| 0 | 102 | CXU | R | A | 0.231 | 0.767 | 3.320 |
| 0 | 102 | CMS | R | A | 0.540 | 0.459 | 0.850 |
| 0 | 102 | CXS | R | A | 0.229 | 0.771 | 3.372 |

## Plot competition-experiment results¶

**Plot enrichment of mutation relative to wildtype in MxA-expressing vs non-expressing cells**

Below we show ratios of relative frequencies in MxA-expressing and non-expressing cells at 10 and 54 hours post-infection for each biological replicate of the competition experiment. The relative frequency is the ratio of the predicted MxA resistance or sensitive mutation frequency (identified by high-throughput deep mutational scanning experiment) relative to the wildtype amino acid frequency at the site at a given timepoint in the competition. We take the ratio of these relative frequencies in MxA-expressing and non-expressing cells, at 10 and 54 hours post-infection. Each NP mutation is then plotted ranked by the magnitude of this quantity at 54 hours post-infection.

In [11]:

```
plotfile = '%s/supp_competitionallsitesreplicates.pdf' % figdir
fig, axes = plt.subplots(nrows=1, ncols=2, figsize=(16, 8))
columns = ['mutation', 'replicate', 'ratio_10hrs', 'ratio_54hrs']

for replicate, ax in zip(replicates, axes):
    ratio = pd.DataFrame(columns=columns)
    for (site, wt, mut, amplicon) in tested_muts:        
        if amplicon in amplicons_nonsyn:      
            CMU = float(freq[(freq.site==site) & (freq.replicate==replicate) & (freq.experiment=='CMU')]['mut_wt_ratio'])
            CXU = float(freq[(freq.site==site) & (freq.replicate==replicate) & (freq.experiment=='CXU')]['mut_wt_ratio'])
            CMS = float(freq[(freq.site==site) & (freq.replicate==replicate) & (freq.experiment=='CMS')]['mut_wt_ratio'])
            CXS = float(freq[(freq.site==site) & (freq.replicate==replicate) & (freq.experiment=='CXS')]['mut_wt_ratio'])
        elif amplicon in amplicons_syn:
            CMU = float(freq[(freq.site==site) & (freq.replicate==replicate) & (freq.experiment=='CMUsyn')]['mut_wt_ratio'])
            CXU = float(freq[(freq.site==site) & (freq.replicate==replicate) & (freq.experiment=='CXUsyn')]['mut_wt_ratio'])
            CMS = float(freq[(freq.site==site) & (freq.replicate==replicate) & (freq.experiment=='CMSsyn')]['mut_wt_ratio'])
            CXS = float(freq[(freq.site==site) & (freq.replicate==replicate) & (freq.experiment=='CXSsyn')]['mut_wt_ratio'])  
            wt, mut = 'D', 'Dsyn'   # change label for synonymous mutations
        else:
            raise ValueError('Amplicon %s not found' % amplicon)
        
        ratio.loc[len(ratio)] = ['%s%s%s' % (wt, site, mut), replicate, CXU/CMU, CXS/CMS]  # ratio of relative frequencies

    # Sort dataframe rows by ratio ofrelative frequencies at 54 hpi. 
    ratio.sort_values(by='ratio_54hrs', inplace=True)
    ratio.reset_index(inplace=True, drop=True)

    x = range(1, len(tested_muts)+1)
    ax.scatter(x[0:9], ratio['ratio_54hrs'][0:9], marker='^', color='b', s=100, clip_on=False)
    ax.scatter(x[0:9], ratio['ratio_10hrs'][0:9], marker='o', color='b', s=100, clip_on=False)
    ax.scatter(x[9], ratio['ratio_54hrs'][9], marker='^', color='k', label='syn54hpi', s=100, clip_on=False)
    ax.scatter(x[9], ratio['ratio_10hrs'][9], marker='o', color='k', label='syn10hpi', s=100, clip_on=False)
    ax.scatter(x[10:], ratio['ratio_54hrs'][10:], marker='^', color='r', s=100, clip_on=False)
    ax.scatter(x[10:], ratio['ratio_10hrs'][10:], marker='o', color='r', s=100, clip_on=False)

    # Axis properties
    spineOffset = {'left': 10, 'bottom': 0}    
    [spine.set_position(('outward',spineOffset[loc])) if loc in ['left','bottom'] else spine.set_color('none') for loc, spine in ax.spines.items() ] 
    ax.xaxis.set(ticks_position = 'bottom')
    ax.yaxis.set(ticks_position = 'left')

    ax.set_xticks(x)
    ax.set_xticklabels(ratio['mutation'], fontsize=20, rotation='vertical')
    ax.set_xlabel('mutation', fontsize=30)

    ax.set_yscale('log')
    ax.yaxis.set_tick_params(labelsize=20, width=3)
    ylabels = ['$\mathregular{10^{-4}}$', '$\mathregular{10^{-3}}$', '$\mathregular{10^{-2}}$', '$\mathregular{10^{-1}}$', '$\mathregular{1}$', '$\mathregular{10^{1}}$']
    ax.set_yticks([1e-4, 1e-3, 1e-2, 1e-1, 1, 1e1])
    ax.set_ylim([1e-4, 1e1])
    ax.set_yticklabels(ylabels, fontsize=20)
    ax.set_title('replicate %s' % replicate, fontsize=30)

    ax.axhline(y=1, linewidth=2, color='gray', linestyle='--', alpha=.4)

axes[0].set_ylabel('enrichment in\nMxA expressing cells', fontsize=30)
artist54hpi = plt.Line2D([0], [0], marker='^', mfc='None', mec='k', mew=2, markersize=10, linewidth=0)
artist10hpi = plt.Line2D([0], [0], marker='o', mfc='None', mec='k', mew=2, markersize=10, linewidth=0)
axes[0].legend((artist54hpi,artist10hpi),('54 hpi', '10 hpi'),bbox_to_anchor=(.3,1), numpoints=1, frameon=False, fontsize=22, handletextpad=0)

plt.tight_layout()
plt.savefig(plotfile)
ShowPDFinline(plotfile, figdir, 800)
```

```
/home/oashenbe/.local/lib/python2.7/site-packages/matplotlib/tight_layout.py:222: UserWarning: tight_layout : falling back to Agg renderer
  warnings.warn("tight_layout : falling back to Agg renderer")
```

Below we show ratios of relative frequencies in MxA-expressing and non-expressing cells at 10 and 54 hours post-infection, where the average mutation frequency has been taken across the biological replicates of the competition experiment.

In [12]:

```
plotfile = '%s/competitionallsites.pdf' % figdir
columns = ['mutation', 'ratio_10hrs', 'ratio_54hrs']
ratio = pd.DataFrame(columns=columns)

for (site, wt, mut, amplicon) in tested_muts:        
    if amplicon in amplicons_nonsyn:      
        CMU = float(meanfreq[(meanfreq.site==site) & (meanfreq.experiment=='CMU')]['mut_wt_ratio'])
        CXU = float(meanfreq[(meanfreq.site==site) & (meanfreq.experiment=='CXU')]['mut_wt_ratio'])
        CMS = float(meanfreq[(meanfreq.site==site) & (meanfreq.experiment=='CMS')]['mut_wt_ratio'])
        CXS = float(meanfreq[(meanfreq.site==site) & (meanfreq.experiment=='CXS')]['mut_wt_ratio'])
    elif amplicon in amplicons_syn:
        CMU = float(meanfreq[(meanfreq.site==site) & (meanfreq.experiment=='CMUsyn')]['mut_wt_ratio'])
        CXU = float(meanfreq[(meanfreq.site==site) & (meanfreq.experiment=='CXUsyn')]['mut_wt_ratio'])
        CMS = float(meanfreq[(meanfreq.site==site) & (meanfreq.experiment=='CMSsyn')]['mut_wt_ratio'])
        CXS = float(meanfreq[(meanfreq.site==site) & (meanfreq.experiment=='CXSsyn')]['mut_wt_ratio'])   
        wt, mut = 'D', 'Dsyn'   # change label for synonymous mutations
    else:
        raise ValueError('Amplicon %s not found' % amplicon)

    ratio.loc[len(ratio)] = ['%s%s%s' % (wt, site, mut), CXU/CMU, CXS/CMS] # ratio of relative frequencies

# Sort dataframe rows by ratio at 54 hpi. 
ratio.sort_values(by='ratio_54hrs', inplace=True)
ratio.reset_index(inplace=True, drop=True)

x = range(1, len(tested_muts)+1)
fig, ax = plt.subplots(figsize=(8, 8))
ax.scatter(x[0:9], ratio['ratio_54hrs'][0:9], marker='^', color='b', s=100, clip_on=False)
ax.scatter(x[0:9], ratio['ratio_10hrs'][0:9], marker='o', color='b', s=100, clip_on=False)
ax.scatter(x[9], ratio['ratio_54hrs'][9], marker='^', color='k', label='syn54hpi', s=100, clip_on=False)
ax.scatter(x[9], ratio['ratio_10hrs'][9], marker='o', color='k', label='syn10hpi', s=100, clip_on=False)
ax.scatter(x[10:], ratio['ratio_54hrs'][10:], marker='^', color='r', s=100, clip_on=False)
ax.scatter(x[10:], ratio['ratio_10hrs'][10:], marker='o', color='r', s=100, clip_on=False)

# Axis properties
spineOffset = {'left': 10, 'bottom': 0}    
[spine.set_position(('outward',spineOffset[loc])) if loc in ['left','bottom'] else spine.set_color('none') for loc, spine in ax.spines.items() ] 
ax.xaxis.set(ticks_position = 'bottom')
ax.yaxis.set(ticks_position = 'left')

ax.set_xticks(x)
ax.set_xticklabels(ratio['mutation'], fontsize=20, rotation='vertical')
ax.set_xlabel('mutation', fontsize=30)

ax.set_yscale('log')
ax.yaxis.set_tick_params(labelsize=20, width=3)
ylabels = ['$\mathregular{10^{-3}}$', '$\mathregular{10^{-2}}$', '$\mathregular{10^{-1}}$', '$\mathregular{1}$', '$\mathregular{10^{1}}$']
ax.set_yticks([1e-3, 1e-2, 1e-1, 1, 1e1])
ax.set_ylim([9e-4, 1e1])
ax.set_yticklabels(ylabels, fontsize=20)
ax.set_ylabel('enrichment in\nMxA expressing cells', fontsize=30)

artist54hpi = plt.Line2D([0], [0], marker='^', mfc='None', mec='k', mew=2, markersize=10, linewidth=0)
artist10hpi = plt.Line2D([0], [0], marker='o', mfc='None', mec='k', mew=2, markersize=10, linewidth=0)
ax.legend((artist54hpi,artist10hpi),('54 hpi', '10 hpi'),bbox_to_anchor=(.3,1), numpoints=1, frameon=False, fontsize=22, handletextpad=0)

ax.axhline(y=1, linewidth=2, color='gray', linestyle='--', alpha=.4)

plt.tight_layout()
plt.savefig(plotfile)
ShowPDFinline(plotfile, figdir, 500)
```

**Plot relative NP mutation frequencies over time**

Below, we plot the relative mutation frequencies over time for replicates of the competition experiment at 10 and 54 hours post infection. The relative frequency is the ratio of the predicted MxA resistance or sensitive mutation frequency to the wildtype amino acid frequency at the site at a given timepoint in the competition experiment.

In [13]:

```
plotfile = '%s/supp_competition.pdf' % figdir
plt.close('all') 
plt.rc('font', family='sans-serif')
xlabels = ['-MxA', '+MxA', '-MxA', '+MxA']
ylabel = 'Frequency'
times = [0, 10, 54]     # hours post-infection
fig, axes = plt.subplots(nrows=11, ncols=2, figsize=(12, 55))  

# Plot amino-acid or codon frequency for a given site depending on mutation type
ax_ind = 0
for (site, wt, mut, amplicon) in tested_muts[2:]:
    values = []
    for replicate in replicates:
        if amplicon in amplicons_nonsyn:
            plotfreq = dict([(experiment, float(freq[(freq.site==site) & (freq.replicate==replicate) & (freq.experiment==experiment)]['mut_wt_ratio'])) for experiment in experiments_nonsyn])
        else:
            plotfreq = dict([(experiment, float(freq[(freq.site==site) & (freq.replicate==replicate) & (freq.experiment==experiment)]['mut_wt_ratio'])) for experiment in experiments_syn])    
            # rename dictionary keys to make plotting easier
            plotfreq['CMU'] = plotfreq.pop('CMUsyn')
            plotfreq['CXU'] = plotfreq.pop('CXUsyn')
            plotfreq['CMS'] = plotfreq.pop('CMSsyn')
            plotfreq['CXS'] = plotfreq.pop('CXSsyn')
            wt, mut = 'D', 'Dsyn'   # change label for synonymous mutations

        # points and lines to connect for 10, 54 hrs post infection
        ax = axes.flatten()[ax_ind]
        ax.plot(times[1:], [plotfreq['CMU'], plotfreq['CMS']], 'b', linewidth=3, marker='s', markersize=15, clip_on=False)    # MDCK-SIAT1
        ax.plot(times[1:], [plotfreq['CXU'], plotfreq['CXS']], 'r', linewidth=3, marker='o', markersize=15, clip_on=False)    # MDCK-SIAT1-MxA

        # points and dashed lines to connect to 0 hrs post infection (target input)
        ax.scatter(times[0], 1, facecolors='none', edgecolors='k', s=150, clip_on=False)
        ax.plot(times[:2], [1, plotfreq['CMU']], 'b--', linewidth=3)
        ax.plot(times[:2], [1, plotfreq['CXU']], 'r--', linewidth=3)        

        values.extend(plotfreq.values())   # store these to get the minimum and maximum y-data for plotting across replicates
        ax_ind +=1

    axes.flatten()[ax_ind-2].set_ylabel('%s%s:%s%s' % (mut, site, wt, site), fontsize=30)

    # Determine range of values to plot relative mutant frequency data from both replicates
    max_val = max(int(math.ceil(math.log10(max(values)))), 1)
    min_val = min(int(math.floor(math.log10(min(values)))), -1)
    ylabels = ['$\mathregular{10^{%s}}$' % val if val else '$\mathregular{1}$' for val in range(min_val, max_val+1)]
    for ax in axes.flatten()[ax_ind-2:ax_ind]:
        ax.axhline(y=1, linewidth=2, color='gray', linestyle='--')
        ax.set_yscale('log')
        ax.set_ylim([10**min_val, 10**max_val])
        ax.set_yticks([10**val for val in range(min_val, max_val+1)])
        ax.yaxis.set_tick_params(labelsize=20, width=3)
        ax.set_yticklabels(ylabels)
        
# Axis properties
spineOffset = {'left': 10, 'bottom': 0}    
for ax in axes.flatten():
    [spine.set_position(('outward',spineOffset[loc])) if loc in ['left','bottom'] else spine.set_color('none') for loc, spine in ax.spines.items() ] 
    ax.xaxis.set(ticks_position = 'bottom')
    ax.yaxis.set(ticks_position = 'left')

    ax.set_xlim([times[0]-1, times[2]+1])
    ax.set_xticks(times)
    ax.xaxis.set_tick_params(width=3)
    ax.set_xticklabels([])
    
axes[0,0].set_title('replicate 1', fontsize=30)
axes[0,1].set_title('replicate 2', fontsize=30)
axes[10,0].set_xticklabels(['target input', '10 hpi', '54 hpi'], fontsize=30, rotation='vertical')
axes[10,1].set_xticklabels(['target input', '10 hpi', '54 hpi'], fontsize=30, rotation='vertical')

axes[0,0].legend(['MDCK-SIAT1', 'MDCK-SIAT1-\nMxA'], bbox_to_anchor=(.9,.9), bbox_transform=plt.gcf().transFigure, frameon=False, numpoints=1, fontsize=22)

# plt.tight_layout()
plt.savefig(plotfile)
ShowPDFinline(plotfile, figdir, 700)
```

Below, we plot the relative mutation frequencies over time for replicates of the competition experiment at 10 and 54 hours post infection for NP site 51 only.

In [14]:

```
plotfile = '%s/competitionsite51.pdf' % figdir
plt.close('all') 
plt.rc('font', family='sans-serif')
site = '51'
xlabels = ['-MxA', '+MxA', '-MxA', '+MxA']
ylabel = 'Frequency'
times = [0, 10, 54]     # hours post-infection
fig, axes = plt.subplots(nrows=2, ncols=2, figsize=(13, 12))

# Plot amino-acid or codon frequency for a given site depending on mutation type
# D51N
for replicate, ax in zip(replicates, (axes[0][0], axes[0][1])):
    plotfreq = dict([(experiment, float(freq[(freq.site==site) & (freq.replicate==replicate) & (freq.experiment==experiment)]['mut_wt_ratio'])) for experiment in experiments_nonsyn])

    # points and lines to connect for 10, 54 hrs post infection
    ax.plot(times[1:], [plotfreq['CMU'], plotfreq['CMS']], 'b', linewidth=3, marker='s', markersize=15, clip_on=False)    # MDCK-SIAT1
    ax.plot(times[1:], [plotfreq['CXU'], plotfreq['CXS']], 'r', linewidth=3, marker='o', markersize=15, clip_on=False)    # MDCK-SIAT1-MxA

    # points and dashed lines to connect to 0 hrs post infection (target input)
    ax.scatter(times[0], 1, facecolors='none', edgecolors='k', s=150, clip_on=False)
    ax.plot(times[:2], [1, plotfreq['CMU']], 'b--', linewidth=3)
    ax.plot(times[:2], [1, plotfreq['CXU']], 'r--', linewidth=3)

# D51Dsyn
for replicate, ax in zip(replicates, (axes[1][0], axes[1][1])):
    plotfreq = dict([(experiment, float(freq[(freq.site==site) & (freq.replicate==replicate) & (freq.experiment==experiment)]['mut_wt_ratio'])) for experiment in experiments_syn]) 

    # points and lines to connect for 10, 54 hrs post infection
    ax.plot(times[1:], [plotfreq['CMUsyn'], plotfreq['CMSsyn']], 'b', linewidth=3, marker='s', markersize=15, clip_on=False)    # MDCK-SIAT1
    ax.plot(times[1:], [plotfreq['CXUsyn'], plotfreq['CXSsyn']], 'r', linewidth=3, marker='o', markersize=15, clip_on=False)    # MDCK-SIAT1-MxA

    # points and dashed lines to connect to 0 hrs post infection (target input)
    ax.scatter(times[0], 1, facecolors='none', edgecolors='k', s=150, clip_on=False)
    ax.plot(times[:2], [1, plotfreq['CMUsyn']], 'b--', linewidth=3)
    ax.plot(times[:2], [1, plotfreq['CXUsyn']], 'r--', linewidth=3)

# Axis properties
spineOffset = {'left': 10, 'bottom': 0}    
for ax in axes.flatten():
    ax.axhline(y=1, linewidth=2, color='gray', linestyle='--')

    [spine.set_position(('outward',spineOffset[loc])) if loc in ['left','bottom'] else spine.set_color('none') for loc, spine in ax.spines.items() ] 
    ax.xaxis.set(ticks_position = 'bottom')
    ax.yaxis.set(ticks_position = 'left')

    ax.set_xlim([times[0]-1, times[2]+1])
    ax.set_xticks(times)
    ax.xaxis.set_tick_params(width=3)
    ax.set_xticklabels([])
    
    ax.set_yscale('log')
    ax.set_ylim([7e-4, 1e1])
    ax.yaxis.set_tick_params(labelsize=20, width=3)
    ax.set_yticklabels([])

axes[0,0].set_title('replicate 1', fontsize=30)
axes[0,1].set_title('replicate 2', fontsize=30)

axes[1,0].set_xticklabels(['target input', '10 hpi', '54 hpi'], fontsize=30, rotation='vertical')
axes[1,1].set_xticklabels(['target input', '10 hpi', '54 hpi'], fontsize=30, rotation='vertical')

axes[0,0].set_ylabel('N51:D51', fontsize=30)
axes[1, 0].set_ylabel('Dsyn51:D51', fontsize=30)

axes[0,1].legend(['MDCK-SIAT1', 'MDCK-SIAT1-\nMxA'], bbox_to_anchor=(1.25,.9), bbox_transform=plt.gcf().transFigure, frameon=False, numpoints=1, fontsize=22)

ylabels = ['$\mathregular{10^{-3}}$', '$\mathregular{10^{-2}}$', '$\mathregular{10^{-1}}$','$\mathregular{1}$', '$\mathregular{10^{1}}$']
axes[0,0].set_yticks([1e-3, 1e-2, 1e-1, 1, 1e1])
axes[1,0].set_yticks([1e-3, 1e-2, 1e-1, 1, 1e1])
axes[0,0].set_yticklabels(ylabels, fontsize=26)
axes[1,0].set_yticklabels(ylabels, fontsize=26)

plt.tight_layout()
plt.savefig(plotfile, bbox_inches='tight')
ShowPDFinline(plotfile, figdir, 700)
```

In [ ]:

```

```
