# Supplementary material for: Deep mutational scanning identifies sites in influenza nucleoprotein that affect viral inhibition by MxA
Source: PLoS Pathog. 2017 Mar 27;13(3):e1006288. doi: 10.1371/journal.ppat.1006288 (PMC5383324; doi:10.1371/journal.ppat.1006288)
Supplement: S1 File — This ZIP file contains the computer code and input data files to perform the analysis described in this paper. (ZIP) [file ppat.1006288.s011.zip › Supplemental_File_1_code/DeepMutationalScanNotebook.html]

DeepMutationalScanNotebook


# Analysis of deep mutational scanning of influenza A/Aichi/1968 (H3N2) nucleoprotein sensitivity to human MxA¶

Orr Ashenberg, 24 August 2016

To run the commands in this notebook, please first install ```Biopython ``` and ```dms\_tools```, and then verify that they can be run in the directory in which you placed this ipython notebook.

---

## Table of contents¶

### Load required modules and raw data¶

Preliminary set-up and load required modules

Download sequencing data from the SRA

### Process and analyze deep sequencing data with `mapmuts`¶

Aligning paired-end reads with ```mapmuts\_makealignments```

Parsing mutation counts with ```mapmuts\_parsecounts```

### Calculate differential selection and make figures¶

Calculate differential selection for each mutation and each site in NP

Correlations between site differential selections and mutation differential selections for biological replicates

Distributions of site differential selections and mutation differential selections

Logo plot of mutation differential selections at each NP site

NP sites or mutations with the greatest differential selection

Mutation differential selections at site 51 for both biological replicates

---

## Load required modules and raw data¶

**Preliminary set-up and load required modules:**

In [1]:

```
import os
basedir = os.getcwd()
if not os.path.isdir('notebook_figures'):
    !mkdir notebook_figures
if not os.path.isdir('FASTQ_files'):
    !mkdir FASTQ_files
figdir = '%s/notebook_figures' % basedir

import matplotlib
print "Using matplotlib version %s" % matplotlib.__version__
matplotlib.use("Pdf")
import numpy as np
import matplotlib.pyplot as plt
import scipy.stats
from operator import itemgetter
import os
import subprocess
import math
import glob
import pandas as pd
import dms_tools.plot
import dms_tools.file_io
import dms_tools.utils
import dms_tools.weblogo
from runscriptfunctions import * # functions to facilitate running parallel jobs on the Fred Hutch scientific computing cluster using `slurm`
```

```
Using matplotlib version 1.5.0
```

Specifications for running `mapmuts` scripts. The only flag that the user may need to change is the `use_sbatch` flag.

In [2]:

```
# Where FASTQ files from SRA will be downloaded
FASTQdir = '%s/FASTQ_files' % basedir

# sbatch submits a batch script to Slurm, a program for sending jobs to a computer cluster among other things. 
# If you do not have sbatch installed, set this flag to False. 
# The most computationally intense activity of this script is running mapmuts_makealignments, 
# which without using sbatch will take only a few hours on a modern computer (Align reads for 14 individual replicate-amplicons, each takes ~30 min). 
use_sbatch = True

# Maximum number of CPUs to try to use at once. If not using sbatch for running mapmuts scripts on a cluster, don't make this bigger than the number of available cores.
max_cpus = 100

# Specify the biological replicates and library amplicons that were sequenced.
replicates = ['1', '2']
amplicons = ['DNA', 'mutDNA', 'vir', 'mutvir', 'mutvir_MS', 'mutvir_MxA', 'mutvir_MxAneg']

# Specify range of nucleotides in the Aichi/1968 NP amplicon to use for aligning reads
generange = (62, 1555) 

# Specify range of residues to consider in mapmuts_countparsedmuts.py
sitesrange = (2, 498)
```

Specifications for running `dms_tools` scripts. Nothing here needs to be changed by the user.

In [3]:

```
# Specify which mutant virus libraries to use as control selection and MxA selection to calculate differential selections
controls = ['mutvir_MS', 'mutvir_MxAneg']
selection = 'mutvir_MxA'

# List of MxA sensitivity mutations. Tuple elements are (site, resistance mutation, sensitivity mutation)
# These mutations were characterized by Mänz, Benjamin, et al. PLoS Pathog 9.3 (2013): e1003279.
lit = [('100', 'V', 'R'), ('283', 'P', 'L'), ('313', 'Y', 'F')]
```

**Download sequencing data from the SRA:**

In [4]:

```
%%capture
samples = {'SRR4048733':'DNA-1',
           'SRR4048734':'vir-1',
           'SRR4048735':'mutDNA-1',
           'SRR4048736':'mutvir-1',
           'SRR4048737':'mutvir_MS-1',
           'SRR4048738':'mutvir_MxA-1',
           'SRR4048739':'mutvir_MxAneg-1',
           'SRR4048740':'DNA-2',
           'SRR4048741':'vir-2',
           'SRR4048742':'mutDNA-2',
           'SRR4048743':'mutvir-2',
           'SRR4048744':'mutvir_MS-2',
           'SRR4048745':'mutvir_MxA-2',
           'SRR4048746':'mutvir_MxAneg-2'}

for (accession, samplename) in samples.items():
    print "Downloading and converting file format for sample %s, which corresponds to accession %s." % (samplename, accession)
    !cd FASTQ_files; wget --no-clobber 'ftp://ftp-trace.ncbi.nih.gov/sra/sra-instant/reads/ByRun/sra/SRR/SRR404/'$accession'/'$accession'.sra'
    !mv "FASTQ_files/"$accession".sra" "FASTQ_files/"$samplename".sra"
    !fastq-dump -O FASTQ_files/ --split-files "FASTQ_files/"$samplename".sra"
```

## Process and analyze deep sequencing data¶

**Aligning paired-end reads with** `mapmuts_makealignments`

In [5]:

```
processes = []    # List of process to run (run in parallel if sbatch=True)
# Dictionary to convert amplicon name for a replicate to appropriate barcoded adapter sequence for trimming barcode from R1 read
R1_trims_d = {  'DNA-1' : 'R1_adapter_AR003.fa',
                'vir-1' : 'R1_adapter_AR025.fa',
                'mutDNA-1' : 'R1_adapter_AR009.fa',
                'mutvir-1' : 'R1_adapter_AR022.fa',
                'mutvir_MS-1' : 'R1_adapter_AR002.fa',
                'mutvir_MxA-1' : 'R1_adapter_AR004.fa',
                'mutvir_MxAneg-1' : 'R1_adapter_AR007.fa',
                'DNA-2' : 'R1_adapter_AR003.fa',
                'vir-2' : 'R1_adapter_AR025.fa',
                'mutDNA-2' : 'R1_adapter_AR009.fa',
                'mutvir-2' : 'R1_adapter_AR022.fa',
                'mutvir_MS-2' : 'R1_adapter_AR002.fa',
                'mutvir_MxA-2' : 'R1_adapter_AR004.fa',
                'mutvir_MxAneg-2' : 'R1_adapter_AR007.fa'
             }

# Specifications for running mapmuts_makealignments
command_d = {'gzipped':'False',
             'applyfilter':'True',
             'minq':'25',
             'generange':'%d %d' % generange,
             'fullgenefile' : '%s/input/Aichi68-NP_amplicon.fa' % basedir,
             'a2file':'%s/input/R2_adapterUniversal_RC.fa' % basedir,
             'maxn':'5',
             'minoverlap':'100',
             'maxrm':'1',
             'maxa1m':'1',
             'maxa2m':'1',
             'maxgenem':'10',
             'upcase':'test',
             'write_unaligned':'True',
            }

for replicate in replicates:
    if not os.path.isdir('replicate-%s' % replicate):
        os.mkdir('replicate-%s' % replicate)
    for amplicon in amplicons:
        if not os.path.isdir('replicate-%s/%s' % (replicate, amplicon)):
            os.mkdir('replicate-%s/%s' % (replicate, amplicon))

        # Specify the R1 adapter a1file for this replicate-amplicon combination
        command_d['a1file'] = '%s/input/%s' % (basedir, R1_trims_d[ amplicon + '-' + replicate ])

        # Specify the fastqfiles for this replicate-amplicon
        command_d['r1files'] = '%s/%s-%s_1.fastq' % (FASTQdir, amplicon, replicate)
        command_d['r2files'] = '%s/%s-%s_2.fastq' % (FASTQdir, amplicon, replicate)

        subdir = 'replicate-%s/%s' % (replicate, amplicon)

        # outfileprefixes are specified as "replicate-amplicon" (ie replicate-1-DNA)
        command_d['outfileprefix'] = subdir.replace('/', '-')
        # samplenames are specified by "replicate, amplicon" (ie replicate-1, DNA)
        command_d['samplename'] = subdir.replace('/', ', ')

        # Add this makealignments.py call to the list of processes.
        # Use *list* to make a copy of *commands* since lists are mutable on subsequent runs
        # of making replicate-amplicon combinations.
        processes.append(multiprocessing.Process(target=RunScriptMapmuts,\
            args=(subdir, "makealignments", 'mapmuts_makealignments.py', list(command_d.items()), use_sbatch, 1)))

# run ALL the makealignments processes
RunProcesses(processes, nmultiruns=max_cpus)
```

```
Running mapmuts_makealignments.py for makealignments in directory replicate-1/DNA...
Running mapmuts_makealignments.py for makealignments in directory replicate-1/mutDNA...
Running mapmuts_makealignments.py for makealignments in directory replicate-1/vir...
Running mapmuts_makealignments.py for makealignments in directory replicate-1/mutvir...
Running mapmuts_makealignments.py for makealignments in directory replicate-1/mutvir_MS...
Running mapmuts_makealignments.py for makealignments in directory replicate-1/mutvir_MxA...
Running mapmuts_makealignments.py for makealignments in directory replicate-1/mutvir_MxAneg...
Running mapmuts_makealignments.py for makealignments in directory replicate-2/DNA...
Running mapmuts_makealignments.py for makealignments in directory replicate-2/mutDNA...
Running mapmuts_makealignments.py for makealignments in directory replicate-2/vir...
Running mapmuts_makealignments.py for makealignments in directory replicate-2/mutvir...
Running mapmuts_makealignments.py for makealignments in directory replicate-2/mutvir_MS...
Running mapmuts_makealignments.py for makealignments in directory replicate-2/mutvir_MxA...
Running mapmuts_makealignments.py for makealignments in directory replicate-2/mutvir_MxAneg...
Successfully completed running mapmuts_makealignments.py for makealignments in directory replicate-1/mutvir.Successfully completed running mapmuts_makealignments.py for makealignments in directory replicate-1/mutvir_MS.Successfully completed running mapmuts_makealignments.py for makealignments in directory replicate-1/mutvir_MxAneg.Successfully completed running mapmuts_makealignments.py for makealignments in directory replicate-1/mutvir_MxA.Successfully completed running mapmuts_makealignments.py for makealignments in directory replicate-1/vir.Successfully completed running mapmuts_makealignments.py for makealignments in directory replicate-1/mutDNA.Successfully completed running mapmuts_makealignments.py for makealignments in directory replicate-1/DNA.Successfully completed running mapmuts_makealignments.py for makealignments in directory replicate-2/mutDNA.Successfully completed running mapmuts_makealignments.py for makealignments in directory replicate-2/DNA.Successfully completed running mapmuts_makealignments.py for makealignments in directory replicate-2/mutvir_MS.Successfully completed running mapmuts_makealignments.py for makealignments in directory replicate-2/vir.Successfully completed running mapmuts_makealignments.py for makealignments in directory replicate-2/mutvir.Successfully completed running mapmuts_makealignments.py for makealignments in directory replicate-2/mutvir_MxA.Successfully completed running mapmuts_makealignments.py for makealignments in directory replicate-2/mutvir_MxAneg.
```

**Parsing mutation counts with** `mapmuts_parsecounts`. The main output is counts of the different nucleotide and codon identities at each NP site for each sequenced library.

In [6]:

```
processes = []    # List of process to run (run in parallel if sbatch=True)
command_d = {'generange':'%d %d' % generange,
             'upcase':'test',
             'r1exclude':'1 2 3 4 5 6 7 8 9 10 11 12 13 14 15',
             'r2exclude':'1 2 3 4 5 6 7 8 9 10 11 12 13 14 15',
             'fullgenefile' : '%s/input/Aichi68-NP_amplicon.fa' % basedir
            }

for replicate in replicates:
    for amplicon in amplicons:
        subdir = 'replicate-%s/%s' % (replicate, amplicon)
        # outfileprefixes are specified as "replicate-amplicon" (ie replicate-1-DNA)
        command_d['outfileprefix'] = subdir.replace('/', '-')
        # samplenames are specified by "replicate, amplicon" (ie replicate-1, DNA)
        command_d['samplename'] = subdir.replace('/', ', ') 
        command_d['alignmentfile'] = '%s/%s/%s_alignments.txt.gz' % (basedir, subdir, command_d['outfileprefix'])
        processes.append(multiprocessing.Process(target=RunScriptMapmuts,args=(subdir, 'parsecounts', 'mapmuts_parsecounts.py', list(command_d.items()), use_sbatch, 1)))

RunProcesses(processes, nmultiruns=max_cpus)
```

```
Running mapmuts_parsecounts.py for parsecounts in directory replicate-1/DNA...
Running mapmuts_parsecounts.py for parsecounts in directory replicate-1/mutDNA...
Running mapmuts_parsecounts.py for parsecounts in directory replicate-1/vir...
Running mapmuts_parsecounts.py for parsecounts in directory replicate-1/mutvir...
Running mapmuts_parsecounts.py for parsecounts in directory replicate-1/mutvir_MS...
Running mapmuts_parsecounts.py for parsecounts in directory replicate-1/mutvir_MxA...
Running mapmuts_parsecounts.py for parsecounts in directory replicate-1/mutvir_MxAneg...
Running mapmuts_parsecounts.py for parsecounts in directory replicate-2/DNA...
Running mapmuts_parsecounts.py for parsecounts in directory replicate-2/mutDNA...
Running mapmuts_parsecounts.py for parsecounts in directory replicate-2/vir...
Successfully completed running mapmuts_parsecounts.py for parsecounts in directory replicate-1/mutvir.
Running mapmuts_parsecounts.py for parsecounts in directory replicate-2/mutvir...
Successfully completed running mapmuts_parsecounts.py for parsecounts in directory replicate-1/mutvir_MS.Successfully completed running mapmuts_parsecounts.py for parsecounts in directory replicate-1/DNA.

Running mapmuts_parsecounts.py for parsecounts in directory replicate-2/mutvir_MS...
Successfully completed running mapmuts_parsecounts.py for parsecounts in directory replicate-1/vir.Successfully completed running mapmuts_parsecounts.py for parsecounts in directory replicate-1/mutDNA.

Running mapmuts_parsecounts.py for parsecounts in directory replicate-2/mutvir_MxA...
Successfully completed running mapmuts_parsecounts.py for parsecounts in directory replicate-1/mutvir_MxAneg.
Running mapmuts_parsecounts.py for parsecounts in directory replicate-2/mutvir_MxAneg...
Successfully completed running mapmuts_parsecounts.py for parsecounts in directory replicate-1/mutvir_MxA.Successfully completed running mapmuts_parsecounts.py for parsecounts in directory replicate-2/mutDNA.Successfully completed running mapmuts_parsecounts.py for parsecounts in directory replicate-2/DNA.Successfully completed running mapmuts_parsecounts.py for parsecounts in directory replicate-2/vir.Successfully completed running mapmuts_parsecounts.py for parsecounts in directory replicate-2/mutvir_MS.Successfully completed running mapmuts_parsecounts.py for parsecounts in directory replicate-2/mutvir.Successfully completed running mapmuts_parsecounts.py for parsecounts in directory replicate-2/mutvir_MxA.Successfully completed running mapmuts_parsecounts.py for parsecounts in directory replicate-2/mutvir_MxAneg.
```

Run `mapmuts_parsesummaryplots` to show summary of mutation rates and error rates for the sequenced amplicons.

In [7]:

```
for replicate in replicates:
    commands = []
    for amplicon in amplicons:
        commands.append(('%s/replicate-%s/%s/replicate-%s-%s' % (basedir, replicate, amplicon, replicate, amplicon), "%s %s" % (replicate, amplicon)))

    plotfileprefix = '%s/replicate-%s/parsesummary' % (basedir, replicate)
    commands.append(('plotfileprefix', plotfileprefix))
    commands.append(('writefracs', 'True'))
    commands.append(('textwritefracs', plotfileprefix))
    commands.append(('pairedcodonplot', 'True'))
    #convert_to_pngs.append('%s_codon_types_and_nmuts.pdf' % plotfileprefix)

    RunScriptMapmuts('replicate-%s' % replicate, 'parsesummaryplots', 'mapmuts_parsesummaryplots.py', commands, False, 1)
    ShowPDFinline('%s_codon_types_and_nmuts.pdf' % plotfileprefix, '%s/replicate-%s' % (basedir, replicate), 600)
```

```
Running mapmuts_parsesummaryplots.py for parsesummaryplots in directory replicate-1...
Successfully completed running mapmuts_parsesummaryplots.py for parsesummaryplots in directory replicate-1.
```

```
Running mapmuts_parsesummaryplots.py for parsesummaryplots in directory replicate-2...
Successfully completed running mapmuts_parsesummaryplots.py for parsesummaryplots in directory replicate-2.
```

**Calculate differential selection for each mutation and each site in NP**

We run `dms_diffselection` on MDCK-SIAT1-MxA vs MDCK-SIAT1 (MxA selection), and on MDCK-SIAT1-MxA-T103A vs MDCK-SIAT1 (control selection), for both biological replicates of the mutant virus library.

In [8]:

```
mutdiffsel_files = []   # collect all the mutdiffsel files in order to have the same y-axis scale on subsequent logo plots of differential selection

# MDCK-SIAT1-MxA vs MDCK-SIAT1 (MxA selection)
control = controls[0]
for replicate in replicates:
    subdir = '%s/replicate-%s' % (basedir, replicate)
    args = ['dms_diffselection', \
            '--pseudocount=10', \
            '--mincounts=0',\
            '--chartype=codon_to_aa', \
            '%s/%s/replicate-%s-%s_codoncounts.txt' % (subdir, control, replicate, control), \
            '%s/%s/replicate-%s-%s_codoncounts.txt' % (subdir, selection, replicate, selection), \
            '%s/replicate-%s_%s_%s_' % (subdir, replicate, selection, control)]
    mutdiffsel_files.append('%s/replicate-%s_%s_%s_mutdiffsel.txt' % (subdir, replicate, selection, control))
    os.system(' '.join(args))

# MDCK-SIAT1-MxA-T103A vs MDCK-SIAT1 (control selection)
for replicate in replicates:
    subdir = '%s/replicate-%s' % (basedir, replicate)
    args = ['dms_diffselection', \
            '--pseudocount=10', \
            '--mincounts=0',\
            '--chartype=codon_to_aa', \
            '%s/%s/replicate-%s-%s_codoncounts.txt' % (subdir, controls[0], replicate, controls[0]), \
            '%s/%s/replicate-%s-%s_codoncounts.txt' % (subdir, controls[1], replicate, controls[1]), \
            '%s/replicate-%s_%s_%s_' % (subdir, replicate, controls[1], controls[0])]
    mutdiffsel_files.append('%s/replicate-%s_%s_%s_mutdiffsel.txt' % (subdir, replicate, controls[1], controls[0]))
    os.system(' '.join(args))
```

We run `dms_merge` to take the average of differential selection values measured for both biological replicates. We calculate the average mutation differential selection and the average site differential selection.

In [9]:

```
# MDCK-SIAT1-MxA vs MDCK-SIAT1 (MxA selection) 
control = controls[0]
meanmutfile = '%s/%s_%s_mean_mutdiffsel.txt' % (basedir, selection, control)
meansitefile = '%s/%s_%s_mean_sitediffsel.txt' % (basedir, selection, control)
files = ['%s/replicate-%s/replicate-%s_%s_%s_mutdiffsel.txt' % (basedir, replicate, replicate, selection, control) for replicate in replicates]
args = ['dms_merge', \
        meanmutfile, \
        'average', files[0], files[1], \
        '--sitediffselfile', meansitefile]
os.system(' '.join(args))
 
# MDCK-SIAT1-MxA-T103A vs MDCK-SIAT1 (control selection) 
meanmutfile = '%s/%s_%s_mean_mutdiffsel.txt' % (basedir, controls[1], controls[0])
meansitefile = '%s/%s_%s_mean_sitediffsel.txt' % (basedir, controls[1], controls[0])
files = ['%s/replicate-%s/replicate-%s_%s_%s_mutdiffsel.txt' % (basedir, replicate, replicate, controls[1], controls[0]) for replicate in replicates]
args = ['dms_merge', \
        meanmutfile, \
        'average', files[0], files[1], \
        '--sitediffselfile', meansitefile]
os.system(' '.join(args))
```

Out[9]:

```
0
```

**Correlations between site differential selections and mutation differential selections for biological replicates**

In [10]:

```
# site differential selection of MDCK-SIAT1-MxA vs MDCK-SIAT1 (MxA selection) 
control = controls[0]
files = ['%s/replicate-%s/replicate-%s_%s_%s_sitediffsel.txt' % (basedir, replicate, replicate, selection, control) for replicate in replicates]
plotfile = '%s/%s_%s_sitediffsel_correlationplot' % (figdir, selection, control)
args = ['dms_correlate', \
        files[0], files[1], plotfile, \
        '--name1', 'replicate 1', '--name2', 'replicate 2', '--plot_title', 'site\ndifferential selection', '--alpha=0.3', '--corr_on_plot']
subprocess.check_call(args)    
#print 'selection vs control ', os.path.basename(files[0]), os.path.basename(files[1]), os.path.basename(plotfile)
ShowPDFinline(plotfile+'.pdf', figdir, 250)

# mutation differential selection of MDCK-SIAT1-MxA vs MDCK-SIAT1 (MxA selection) 
files = ['%s/replicate-%s/replicate-%s_%s_%s_mutdiffsel.txt' % (basedir, replicate, replicate, selection, control) for replicate in replicates]
plotfile = '%s/%s_%s_mutdiffsel_correlationplot' % (figdir, selection, control)
args = ['dms_correlate', \
        files[0], files[1], plotfile, \
        '--name1', 'replicate 1', '--name2', 'replicate 2', '--plot_title', 'mutation\ndifferential selection', '--corr_on_plot']
subprocess.check_call(args)
#print 'selection vs control ', os.path.basename(files[0]), os.path.basename(files[1]), os.path.basename(plotfile)
ShowPDFinline(plotfile+'.pdf', figdir, 250)
```

**Distributions of site differential selections and mutation differential selections**

We show the distributions for site differential selection and mutation differential selections for MxA selection and control selection. These values are shown for both biological replicates. In addition we take the mean of these values across the two biological replicates. With the green dots, we mark the NP mutations at sites 100, 283, and 313 characterized by Mänz, Benjamin, et al. PLoS Pathog 9.3 (2013): e1003279.

In [11]:

```
def randjitter(x):
    '''This function is used to help visualize the location of NP mutations at sites 100, 283, and 313,
    which would otherwise overlap in the control selection for mutation differential selection.'''
    jitter = np.array([-.05, 0, .05])
    np.random.shuffle(jitter)
    return x + jitter

plt.close('all')
# List of sitediffsel and mutdiffsel files to display distributions for
sitediffsel_files = ['%s/replicate-%s/replicate-%s_mutvir_MxA_mutvir_MS_sitediffsel.txt' % (basedir, replicates[0], replicates[0]), \
                    '%s/replicate-%s/replicate-%s_mutvir_MxAneg_mutvir_MS_sitediffsel.txt' % (basedir, replicates[0], replicates[0]), \
                    '%s/replicate-%s/replicate-%s_mutvir_MxA_mutvir_MS_sitediffsel.txt' % (basedir, replicates[1], replicates[1]), \
                    '%s/replicate-%s/replicate-%s_mutvir_MxAneg_mutvir_MS_sitediffsel.txt' % (basedir, replicates[1], replicates[1]), \
                    '%s/mutvir_MxA_mutvir_MS_mean_sitediffsel.txt' % basedir, \
                    '%s/mutvir_MxAneg_mutvir_MS_mean_sitediffsel.txt' % basedir]

mutdiffsel_files = ['%s/replicate-%s/replicate-%s_mutvir_MxA_mutvir_MS_mutdiffsel.txt' % (basedir, replicates[0], replicates[0]), \
                    '%s/replicate-%s/replicate-%s_mutvir_MxAneg_mutvir_MS_mutdiffsel.txt' % (basedir, replicates[0], replicates[0]), \
                    '%s/replicate-%s/replicate-%s_mutvir_MxA_mutvir_MS_mutdiffsel.txt' % (basedir, replicates[1], replicates[1]), \
                    '%s/replicate-%s/replicate-%s_mutvir_MxAneg_mutvir_MS_mutdiffsel.txt' % (basedir, replicates[1], replicates[1]), \
                    '%s/mutvir_MxA_mutvir_MS_mean_mutdiffsel.txt' % basedir, \
                    '%s/mutvir_MxAneg_mutvir_MS_mean_mutdiffsel.txt' % basedir]

keys = ('selection 1', 'control 1', 'selection 2', 'control 2', 'selection', 'control')
xlabels = ('MxA', 'control')*3
axespos = np.arange(6)
pos = [1, 1.6, 2.2, 2.8]

# Read mutsiffsel and sitediffsel files
mutdiffsel = {}
sitediffsel = {}
for (mutdiffsel_file, sitediffsel_file, key) in zip(mutdiffsel_files, sitediffsel_files, keys):
    mutdiffsel[key] = pd.read_csv(mutdiffsel_file)
    sitediffsel[key] = pd.read_csv(sitediffsel_file)

##################
# Site differential selection plots 
##################
plt.close('all')
fig, axes = plt.subplots(nrows=1, ncols=2, figsize=(13,6), gridspec_kw = {'width_ratios':[2, 1]})
fig.subplots_adjust(wspace=0.3)   # increase space between both subplots

plots = [sitediffsel[key]['abs_diffsel'] for key in keys]

# Axis properties
for ax in axes:
    spineOffset = {'left': 0, 'bottom': 0}    
    [spine.set_position(('outward',spineOffset[loc])) if loc in ['left','bottom'] else spine.set_color('none') for loc, spine in ax.spines.items() ] 
    ax.xaxis.set(ticks_position = 'none')
    ax.yaxis.set(ticks_position = 'left')
    ax.tick_params(axis='y', labelsize=25)
    ax.set_ylim([0,21])         # manually set by range in data
axes[0].set_ylabel('site\ndifferential selection', fontsize=30) 

# Individual replicates
violin_parts = axes[0].violinplot(plots[:4], pos, points=100, widths=0.4, showmeans=False, showmedians=True, showextrema=True, bw_method="scott")
violin_parts['cmedians'].set_color('black'); violin_parts['cmins'].set_color('black'); violin_parts['cmaxes'].set_color('black'); violin_parts['cbars'].set_color('black')
axes[0].set_xticks(pos) 
axes[0].set_xlim([pos[0]-.3,pos[-1]+.3])
axes[0].set_xticklabels(xlabels[:4], fontsize=24, rotation=0)

# Mean of replicates
violin_parts = axes[1].violinplot(plots[4:], pos[:2], points=100, widths=0.4, showmeans=False, showmedians=True, showextrema=True, bw_method="scott")
violin_parts['cmedians'].set_color('black'); violin_parts['cmins'].set_color('black'); violin_parts['cmaxes'].set_color('black'); violin_parts['cbars'].set_color('black')
axes[1].set_xticks(pos[:2]) 
axes[1].set_xlim([pos[0]-.3,pos[1]+.3])
axes[1].set_xticklabels(xlabels[4:], fontsize=24, rotation=0)

# Literature mutations mapped onto mean sitediffsel distributions for selection vs control and for control vs control
diffsel = sitediffsel['selection']
axes[1].scatter([pos[0]]*len(lit), [float(diffsel.loc[diffsel['site']==int(site)]['abs_diffsel']) for (site, aa_resist, aa_sensitive) in lit], s=50, color='Green')
diffsel = sitediffsel['control']
# a random jitter is applied to the x-values to help display the NP mutations at sites 100,283,313
axes[1].scatter(randjitter([pos[1]]*len(lit)), [float(diffsel.loc[diffsel['site']==int(site)]['abs_diffsel']) for (site, aa_resist, aa_sensitive) in lit], s=50, color='Green')

# Add space to bottom of plot in order to have space for text label below the x-axis
fig.subplots_adjust(bottom=0.2)
ypos = axes[0].get_ylim()[0] - 0.16*(axes[0].get_ylim()[1] - axes[0].get_ylim()[0])
axes[0].text((pos[0]+pos[1])/2, ypos, 'replicate 1', horizontalalignment='center', fontsize=30)
axes[0].text((pos[2]+pos[3])/2, ypos, 'replicate 2', horizontalalignment='center', fontsize=30)
ypos = axes[1].get_ylim()[0] - 0.16*(axes[1].get_ylim()[1] - axes[1].get_ylim()[0])
axes[1].text((pos[0]+pos[1])/2, ypos, 'mean', horizontalalignment='center', fontsize=30)

plotfile = '%s/distribution_sitediffsel.pdf' % figdir
plt.savefig(plotfile)
ShowPDFinline(plotfile, figdir, 800)

##################
# Mutation differential selection plots 
##################
fig, axes = plt.subplots(nrows=1, ncols=2, figsize=(13,6), gridspec_kw = {'width_ratios':[2, 1]})
fig.subplots_adjust(wspace=0.3)   # increase space between both subplots

# Omit NaN values when making violin plots
plots = [[x for x in mutdiffsel[key]['diffsel'] if ~np.isnan(x)] for key in keys]   

# Axis properties
for ax in axes:
    spineOffset = {'left': 0, 'bottom': 0}    
    [spine.set_position(('outward',spineOffset[loc])) if loc in ['left','bottom'] else spine.set_color('none') for loc, spine in ax.spines.items() ] 
    ax.xaxis.set(ticks_position = 'none')
    ax.yaxis.set(ticks_position = 'left')
    ax.tick_params(axis='y', labelsize=25)
axes[0].set_ylim([-8,4])         # manually set by range in data
axes[1].set_ylim([-4,2])         # manually set by range in data
axes[0].set_ylabel('mutation\ndifferential selection', fontsize=30) 

# Individual replicates
violin_parts = axes[0].violinplot(plots[:4], pos, points=100, widths=0.4, showmeans=False, showmedians=True, showextrema=True, bw_method="scott")
violin_parts['cmedians'].set_color('black'); violin_parts['cmins'].set_color('black'); violin_parts['cmaxes'].set_color('black'); violin_parts['cbars'].set_color('black')
axes[0].set_xticks(pos) 
axes[0].set_xlim([pos[0]-.3,pos[-1]+.3])
axes[0].set_xticklabels(xlabels[:4], fontsize=24, rotation=0)

# Mean of replicates
violin_parts = axes[1].violinplot(plots[4:], pos[:2], points=100, widths=0.4, showmeans=False, showmedians=True, showextrema=True, bw_method="scott")
violin_parts['cmedians'].set_color('black'); violin_parts['cmins'].set_color('black'); violin_parts['cmaxes'].set_color('black'); violin_parts['cbars'].set_color('black')
axes[1].set_xticks(pos[:2]) 
axes[1].set_xlim([pos[0]-.3,pos[1]+.3])
axes[1].set_xticklabels(xlabels[4:], fontsize=24, rotation=0)

# Literature mutations mapped onto mean mutdiffsel distributions for selection vs control and for control vs control
diffsel = mutdiffsel['selection']
axes[1].scatter([pos[0]]*len(lit), [float(diffsel[(diffsel['site']==int(site)) & (diffsel['mut']==aa_sensitive)]['diffsel']) for (site, aa_resist, aa_sensitive) in lit], s=50, color='Green')
diffsel = mutdiffsel['control']
axes[1].scatter([pos[1]]*len(lit), [float(diffsel[(diffsel['site']==int(site)) & (diffsel['mut']==aa_sensitive)]['diffsel']) for (site, aa_resist, aa_sensitive) in lit], s=50, color='Green')

# Add space to bottom of plot in order to have space for text label below the x-axis
fig.subplots_adjust(bottom=0.2)
ypos = axes[0].get_ylim()[0] - 0.16*(axes[0].get_ylim()[1] - axes[0].get_ylim()[0])
axes[0].text((pos[0]+pos[1])/2, ypos, 'replicate 1', horizontalalignment='center', fontsize=30)
axes[0].text((pos[2]+pos[3])/2, ypos, 'replicate 2', horizontalalignment='center', fontsize=30)
ypos = axes[1].get_ylim()[0] - 0.16*(axes[1].get_ylim()[1] - axes[1].get_ylim()[0])
axes[1].text((pos[0]+pos[1])/2, ypos, 'mean', horizontalalignment='center', fontsize=30)

plotfile = '%s/distribution_mutdiffsel.pdf' % figdir
plt.savefig(plotfile)
ShowPDFinline(plotfile, figdir, 800)
```

**Logo plot of mutation differential selections at each NP site**

Logo plots are made for differential preferences in the MxA selection condition. Plots are made for both the individual biological replicates and the mean of differential preferences across the replicates.

In [12]:

```
# MDCK-SIAT1-MxA vs MDCK-SIAT1 (MxA selection) individual replicates
control = controls[0]
for replicate in replicates:
    subdir = '%s/replicate-%s' % (basedir, replicate)
    logofile = '%s/replicate-%s_%s_%s_diffsel_logoplot.pdf' % (figdir, replicate, selection, control)
    args = ['dms_logoplot', \
            '%s/replicate-%s_%s_%s_mutdiffsel.txt' % (subdir, replicate, selection, control), \
            logofile, \
             '--nperline=115', \
             '--diffselheight %s' % (' '.join(mutdiffsel_files))]
    os.system(' '.join(args))

# mean selection vs control
logofile = '%s/%s_%s_mean_diffsel_logoplot.pdf' % (figdir, selection, control)
args = ['dms_logoplot', \
        '%s/%s_%s_mean_mutdiffsel.txt' % (basedir, selection, control), \
        logofile, \
         '--nperline=56', \
         '--diffselheight %s' % (' '.join(mutdiffsel_files))]
os.system(' '.join(args))
ShowPDFinline(logofile, figdir, 800)
```

**NP sites or mutations with the greatest differential selection**

**Nucleoprotein sites with greatest mean site differential selection**

Make a logo plot showing the sites with the greatest site differential selection. We look at those sites whose site differential selection in the MxA selection is greater than that in the control selection.

For displaying the logos at each site, a dummy site with differential selection values of 0 is placed between each of the sites of interest. This helps space out the data at each site.

In [13]:

```
# Find sites with greatest site differential selection
meanfile = '%s/mutvir_MxA_mutvir_MS_mean_sitediffsel.txt' % basedir
controlmeanfile = '%s/mutvir_MxAneg_mutvir_MS_mean_sitediffsel.txt' % basedir
diffsel = pd.read_csv(meanfile)
controldiffsel = pd.read_csv(controlmeanfile)

# Find the maximum abs_diffsel in control selection, and use that as a cutoff when looking at abs_diffsel values in MxA selection
max_controldiffsel = controldiffsel['abs_diffsel'][0]   
numtop = len(diffsel[diffsel.abs_diffsel>max_controldiffsel]) # The number of abs_diffsel values greater than a cutoff, the diffsel dataframe is ordered by abs_diffsel magnitude
greatest_sitediffsel = list(diffsel['site'][0:numtop])
print 'The maximum control selection sitediffsel is at site %s with value %.2f, %s sites in MxA selection are greater than this' % (controldiffsel['site'][0], max_controldiffsel, numtop)
print ' '.join([str(i) for i in greatest_sitediffsel])

# Make a dataframe that only has the sites with the greatest site differential selection, and interspersed with 'dummy' sites
# that do not contain actual differential selection data, this allows for spacing when displaying the logo plot
mutdiffselfile = '%s/mutvir_MxA_mutvir_MS_mean_mutdiffsel.txt' % basedir
mutdiffsel = pd.read_csv(mutdiffselfile)
subset = pd.DataFrame(columns=mutdiffsel.columns) 
dummysite = 500  # site that will be spacer between sites of interest, numbering starts past NP numbering which ends at 498
sites = []
for site in greatest_sitediffsel:
    for row in mutdiffsel[mutdiffsel.site==site].itertuples(): # iterate over all rows in dataframe for this specific site: tuple(index, coulmn values)
        subset = subset.append(pd.DataFrame([row[1:]], columns=mutdiffsel.columns))
    for aa in dms_tools.aminoacids:
        subset = subset.append(pd.DataFrame([[dummysite, 'A', aa, 0.0]], columns=mutdiffsel.columns))   # not actual data
    sites.extend([int(site), dummysite])
    dummysite += 1
subset['site'] = subset['site'].apply(lambda x: int(x))
subset.fillna(0.0, inplace=True) # set diffsel to zero for any missing (NaN) values

# Dictionary where key is site, and value is another dictionary 
# That dictionary is keyed by mutation, and value is differential selection for mutation at that site.
# This gets passed to the dms_tools.weblogo.LogoPlot function
data = {}  
logofile = '%s/greatestsitediffsel_logoplot.pdf' % figdir
for r in sites:
    data[str(r)] = subset.loc[subset['site'] == r].set_index('mut')['diffsel'].to_dict()
sites = [str(r) for r in sites] 
dms_tools.weblogo.LogoPlot(sites, 'diffsel', data, logofile, 90, numberevery=2, allowunsorted=True, ylimits=(-20, 5), relativestackheight=3)
ShowPDFinline(logofile, figdir, 900)
```

```
The maximum control selection sitediffsel is at site 23 with value 8.40, 29 sites in MxA selection are greater than this
51 283 23 12 4 85 81 434 466 18 21 294 433 309 41 89 105 50 28 459 14 202 83 424 84 467 399 10 80
```

**Nucleoprotein mutations with greatest mean mutation differential selection**

We show the NP mutations whose mutation differential selection in the MxA selection is outside the range of the mutation differential selection in the control selection. This means the mutation differential selection in the MxA selection is either greater than the maximum in the control selection or less than the minimum in the control selection.

In [14]:

```
# Find the maximum and minimum mutation differential selection in the control selection.
# Use these as cutoffs when looking at mutation differential selection in MxA selection.
control = controls[0]
meanfile = '%s/%s_%s_mean_mutdiffsel.txt' % (basedir, selection, control)
controlmeanfile = '%s/mutvir_MxAneg_mutvir_MS_mean_mutdiffsel.txt' % basedir
diffsel = pd.read_csv(meanfile)
controldiffsel = pd.read_csv(controlmeanfile)

max_controldiffsel = controldiffsel['diffsel'][0]
min_controldiffsel = min(controldiffsel['diffsel'])
diffsel_above_control = diffsel[(diffsel['diffsel']>max_controldiffsel) | (diffsel['diffsel']<min_controldiffsel)]
diffsel_above_control['abs_diffsel'] = np.absolute(diffsel_above_control['diffsel'])
diffsel_above_control = diffsel_above_control.sort_values('abs_diffsel', ascending=False)
print 'The maximum and minimum control selection mutdiffsel are %.2f and %.2f' % (max_controldiffsel, min_controldiffsel)
print ' '.join([str(i) for i in diffsel_above_control['site']])

fig, ax = plt.subplots(tight_layout=True, figsize=(12,6))
width = 0.35
ind = np.arange(len(diffsel_above_control))*2*width
rects = ax.bar(ind, diffsel_above_control['diffsel'], .3, color='k')  # mutation differential selection
plt.axhline(linewidth=1, color='k')

# Axis properties
spineOffset = {'left': 0, 'bottom': 0}    
[spine.set_position(('outward',spineOffset[loc])) if loc in ['left','bottom'] else spine.set_color('none') for loc, spine in ax.spines.items() ] 
ax.xaxis.set(ticks_position = 'bottom')
ax.yaxis.set(ticks_position = 'left')

# Set labels and ticks
xlabels = [''.join(map(str, label)) for label in diffsel_above_control[['wt','site','mut']].values]  # make xlabels from 3 columns (wt,site,mut) of dataframe
ax.set_xticklabels(xlabels, fontsize=20 )
ax.set_xlabel('NP mutation', fontsize=30)
ax.set_xticks(ind+width/2)
plt.xticks(rotation=90)
ax.set_xlim([ind[0]-width/2, ind[-1]+3*width/2])
ax.yaxis.set_tick_params(labelsize=20)
ax.set_ylabel('mutation\ndifferential selection', fontsize=30)

plotfile = '%s/greatestmutdiffsel.pdf' % figdir
plt.savefig(plotfile)
ShowPDFinline(plotfile, figdir, 800)
```

```
The maximum and minimum control selection mutdiffsel are 1.34 and -1.66
14 30 466 43 283 39 59 21 25 51 51 382 446 51 12 51 14 245 16 4 474 181 155 378 283 85 18 294 81 384 232 214 467 102
```

```
/home/oashenbe/.local/lib/python2.7/site-packages/ipykernel/__main__.py:12: SettingWithCopyWarning: 
A value is trying to be set on a copy of a slice from a DataFrame.
Try using .loc[row_indexer,col_indexer] = value instead

See the caveats in the documentation: http://pandas.pydata.org/pandas-docs/stable/indexing.html#indexing-view-versus-copy
```

**Mutation differential selections at site 51 for both biological replicates**

In [15]:

```
# Correlation between replicate measurements of mutation differential selection values at site 51
control = controls[0]
files = ['%s/replicate-%s/replicate-%s_%s_%s_mutdiffsel.txt' % (basedir, replicate, replicate, selection, control) for replicate in replicates]
plotfile = '%s/site51_%s_%s_mutdiffsel_correlationplot.pdf' % (figdir, selection, control)

diffsel1 = pd.read_csv(files[0])
diffsel2 = pd.read_csv(files[1])
diffsel_reps = pd.merge(diffsel1, diffsel2, on=['site', 'wt', 'mut'])
xvalues = diffsel_reps[(diffsel_reps.site == 51)]['diffsel_x']
yvalues = diffsel_reps[(diffsel_reps.site == 51)]['diffsel_y']
new_xvalues, new_yvalues = zip(*[(x,y) for x,y in zip(xvalues, yvalues) if not math.isnan(x) and  not math.isnan(y)])  # remove NaN values when calculating correlation

plt.close('all')
fig, ax = plt.subplots(tight_layout=True, figsize=(6,6))
ax.scatter(new_xvalues, new_yvalues, color='k', s=140)

# Axis properties
spineOffset = {'left': 0, 'bottom': 0}    
[spine.set_position(('outward',spineOffset[loc])) if loc in ['left','bottom'] else spine.set_color('none') for loc, spine in ax.spines.items() ] 
ax.xaxis.set(ticks_position = 'bottom')
ax.yaxis.set(ticks_position = 'left')

# Set labels and ticks
ax.set_xlabel('replicate 1', fontsize=30)
ax.xaxis.set_tick_params(labelsize=30)
ax.set_xticks([-2, -1, 0])
ax.set_xlim([-2.9, 0.5])

ax.set_ylabel('replicate 2', fontsize=30)
ax.yaxis.set_tick_params(labelsize=30)
ax.set_yticks([-2, -1, 0])
ax.set_ylim([-2.9, 0.5])

(r, p) = scipy.stats.pearsonr(new_xvalues, new_yvalues)
text = '$R=%.2f$\n$P=%.4f$' % (r, p)
ax.text(0.05, 0.96, text, horizontalalignment='left', verticalalignment='top', transform=ax.transAxes, size=30)
fig.savefig(plotfile)
ShowPDFinline(plotfile, figdir, 300)
```
